# Responsive Magnetic Polymer Nanocomposites through Thermal-Induced Structural Reorganization

**DOI:** 10.1021/acsnano.4c14311

**Published:** 2025-02-06

**Authors:** Qing Chen, Roman Furrer, Loghman Jamilpanah, Andrei Chumakov, Yusuf Bulut, Constantin Harder, Peter Müller-Buschbaum, Stephan V. Roth, Artur Braun

**Affiliations:** † Spallation Neutron Source Science Center, 523803 Dongguan, China; ‡ Institute of High Energy Physics, Chinese Academy of Science, 100049 Beijing, China; § Laboratory for High Performance Ceramics, Empa, Swiss Federal Laboratories for Materials Science and Technology, 8600 Dübendorf, Switzerland; ∥ Transport at Nanoscale Interfaces Laboratory, Empa, Swiss Federal Laboratories for Materials Science and Technology, 8600 Dübendorf, Switzerland; ⊥ Magnetic and Functional Thin Films Laboratory, Empa, Swiss Federal Laboratories for Materials Science and Technology, 8600 Dübendorf, Switzerland; # Deutsches Elektronen-Synchrotron, 22607 Hamburg, Germany; ∇ TUM School of Natural Sciences, Department of Physics, Chair for Functional Materials, Technical University of Munich, 85748 Garching, Germany; ○ Department of Fiber and Polymer Technology, KTH Royal Institute of Technology, 10044 Stockholm, Sweden

**Keywords:** structural reorganization, polymer nanocomposite, healing, welding, modular assembly

## Abstract

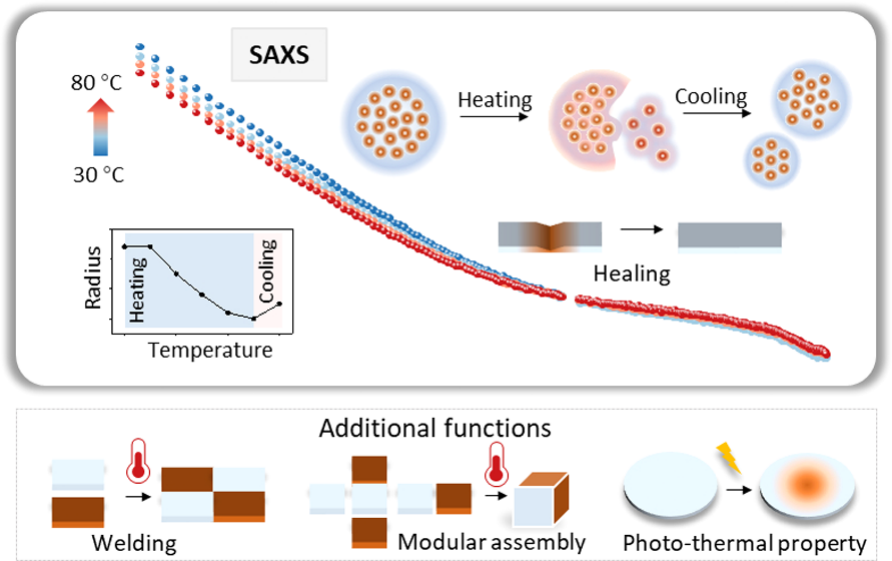

Polymer nanocomposites (PNCs), which feature a hybrid network of soft polymers filled with nanoparticles, hold promise for application in soft robots due to their tunable physiochemical properties. Under certain environmental conditions, PNCs undergo stimuli-responsive structural rearrangement and transform the energy of the ambient environment into diverse uses, for example, repairing the injuries and reconfiguring the shapes of the materials. We develop PNCs with the ability of thermal-responsive restructuring by the stepwise assembly of functional components, including magnetite nanoparticles, silylated cellulose, and polydimethylsiloxane. We investigate the dynamic changes of the nano- and submicron structure of the magnetic PNCs upon the stimulation of heating based on a combined analytical approach: using dynamic mechanical analysis to interpret the viscoelastic properties of the PNC and in situ small-angle X-ray scattering to quantify the clustering of NPs. Based on these results, we formulate a structural model for the heating-induced evolution of the nano- to submicrometer assemblies in the magnetic PNC. Moreover, thermal-induced restructuring of magnetic PNCs leads to additional favorable functions, such as the abilities of healing, welding, reprocessing, and responses to photo and magneto stimuli. Our design provides a versatile means to develop responsive PNCs for applications in soft robots, sensors, and actuators.

The application of soft robots relies on the development of stimuli-responsive materials, which can be activated in response to light, humidity, and magnetic fields.^1^ Stimuli-responsive materials have attracted wide interest owing to their switchable shape,^2^ volume,^3^ and the underlying physiochemical properties upon stimulation.^4^ Within the variety of stimuli-responsive materials, polymer nanocomposites (PNCs) have emerged as a promising class of materials due to their unique combination of a dynamic polymer matrix and functional nanofillers.^5,6^ For example, when magnetite nanoparticles (Fe_3_O_4_ NPs) are selected as fillers, the as-obtained magnetic PNC can be actuated from the spatiotemporal interactions between the applied magnetic field and the redistributed NPs.^7,8^ Great efforts have been devoted to developing stimuli-responsive PNCs with dynamic 3D structures in order to achieve complex modes of deformation. However, the nanofillers are usually physically confined in the polymer matrix after the manufacturing process,^9^ and the soft polymer matrix is normally prone to physical damages such as scratches and fractures. Therefore, most of the current stimuli-responsive PNCs suffer from a lack of healing ability, which is attributed to the constrained assemblies of NPs by the static structural network of the PNC.

Thus, there is a demand for developing stimuli-responsive PNCs with dynamic structural properties in order to prepare these soft materials for adaptive and durable applications.^10^ To date, the strategies for achieving structural rearrangement of PNCs are either to vary the physical arrangement of the fillers and/or that of the polymeric matrix.^11^ Here, structural rearrangement refers to the ability to reconfigure the size and distribution of the nano- or microstructures, which, in the case of stimuli-responsive materials, could enhance the efficiency and adaptability of the as-fabricated actuators. Recently, a series of stimuli-responsive PNCs with the remarkable ability of structural rearrangement has been developed. The polymer matrix in which the nanofillers are embedded typically includes shape memory polymer,^12,13^ thermally responsive polymer,^14^ and liquid crystal elastomer,^15^ which can be activated under specific stimuli and initiate the restructuring of the PNCs. Moreover, the structural rearrangement of PNCs powered by environmental energy sources offers great potential in reconfiguring the macroscopic properties of the composites and gives rise to the possibilities of modular assembly,^16^ actuation deformation,^14,17^ and healing/repairing functions,^18^ or a combination of them.^19^ Specifically, for magneto-responsive PNCs, compared to the commonly used restructuring strategies for PNCs, such as solvent annealing^20^ and induction heating,^21^ moderate heating has been demonstrated as an effective way to reorganize the polymer matrix in order to refine the nano- to microstructures of the NP assemblies without compromising the mechanical properties of the matrix.^18,22^ However, despite the rapid development in magneto-responsive PNCs, it remains a challenge to decipher the mechanisms of structural rearrangement of the PNCs acting at various spatial dimensions.

Compared to the microscopic techniques for observing magnetic PNCs, such as SEM and TEM, which typically collect the structural information at a localized area, small-angle X-ray scattering (SAXS) is a noninvasive technique, which probes the structural information with a much larger statistical significance, and could be easily combined with an in situ environment control module for detecting the continuous structural evolution of the stimuli-responsive PNCs triggered by certain environmental stimuli.^23−25^ Therefore, we study the influence of heating on the structural rearrangement of a magnetic PNC by three analytical techniques targeting different spatial dimensions with temperature control, in which (1) the thermal energy absorption/dissipation abilities are detected by differential scanning calorimetry (DSC), (2) the viscoelastic properties at the macroscale are probed by dynamic mechanical analysis (DMA), and (3) the structural evolution of the magnetic NP assemblies and the polymer matrix at the nano- to submicron scale is studied by SAXS. This strategy allows us to correlate the thermal energy inputs with the structural rearrangement of the composite and propose a model describing the structural evolution of the PNC when being heated.

In this work, we construct a magnetic PNC with the capability of thermally induced structural rearrangement by embedding magnetite nanoparticles (Fe_3_O_4_ NPs) into the dynamic polymer matrix of silylated cellulose (Si-CNC) and polydimethylsiloxane (PDMS). In the following, we will call this the “MCP film”. Due to the thermal responsiveness of Fe_3_O_4_ NPs and the dynamic hydrogen bonding among Si-CNC fibrils, the nano- and microstructures of the MCP film can be reconfigured upon heating. By resolving the morphology, viscoelasticity, and heat-absorbing ability of the MCP film, we detect that the film rearranges through the solid–liquid transition of the encapsulating polymer matrix without altering the intrinsic magnetic properties of embedded particles. Benefiting from the supramolecular cross-linked network of the polymer matrix and the Fe_3_O_4_ NPs, the heat-induced structural rearrangement also leads to additional advantages desirable for the next-generation soft actuators, including the capabilities of healing, welding, modular assembly, and photothermal conversion, which can be used for diversified forms of stimuli-responsive actuation with custom designs.

## Results and Discussion

### Design and the Multiscale Assembly of the Polymer Nanocomposite

**Figure 1:**
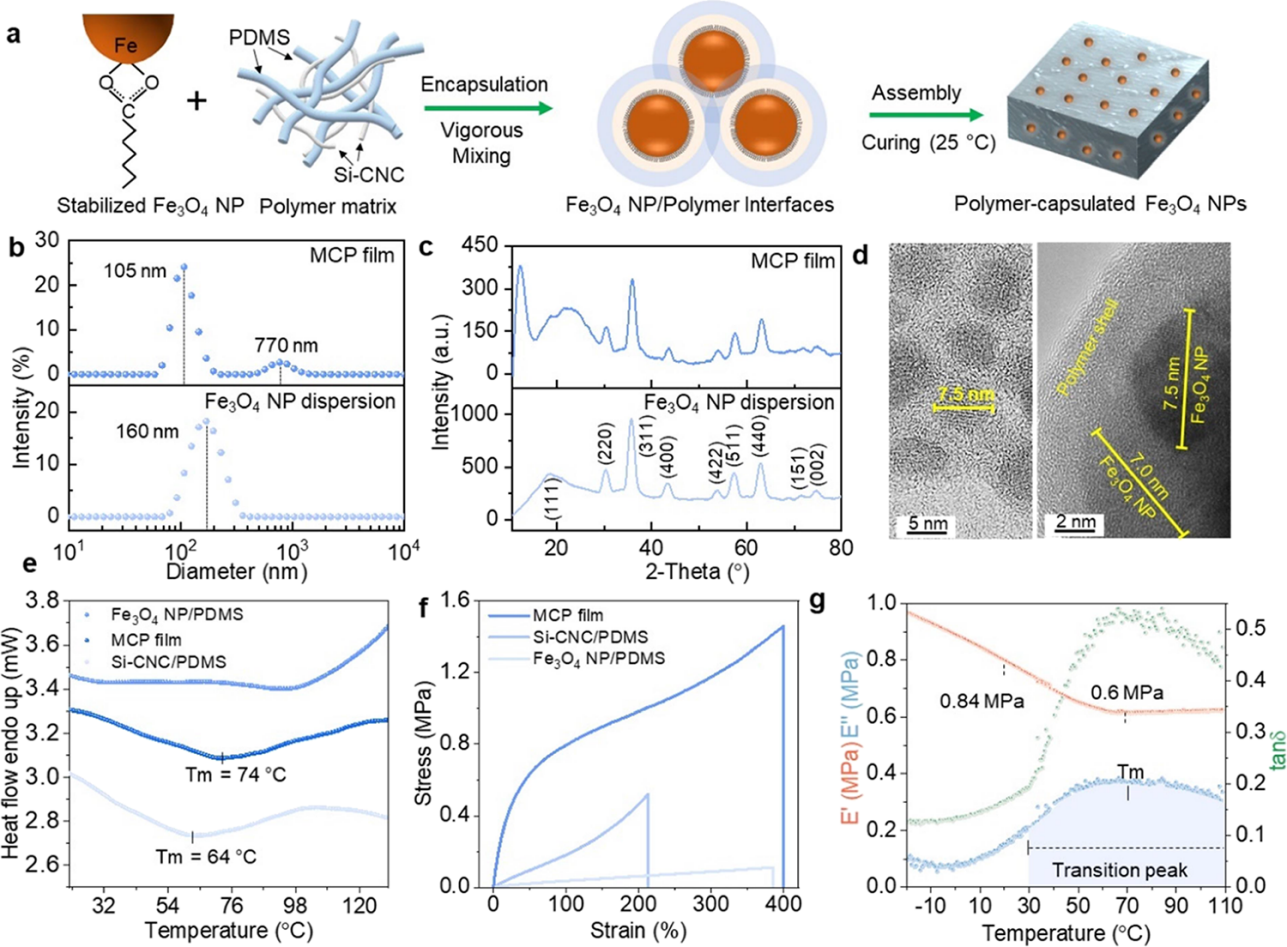
Design and fabrication of the thermal-responsive polymer nanocomposite. (a) Schematic illustration of the proposed assembly of the Fe_3_O_4_NP/Si-CNC/PDMS (MCP) film through the dynamic interactions among Fe_3_O_4_ NPs stabilized by oleic acid and the polymer matrix of Si-CNC and PDMS. (b) Particle size distribution of the Fe_3_O_4_ NPs in water dispersion (bottom panel) and in a mixture of Si-CNC and PDMS (top panel) at 30 °C. (c) XRD analysis of Fe_3_O_4_ NPs (bottom panel) and the MCP film (top panel). (d) TEM micrographs of the Fe_3_O_4_ NPs in an aqueous dispersion (left) and embedded in the mixture of Si-CNC/PDMS (right). (e) Melting temperature of the MCP film detected by DSC analysis. (f, g) Tensile stretching studies (f) and temperature-controlled DMA studies (g) of the MCP film.

Due to the attractive interactions among Fe_3_O_4_ NPs, most of the strategies for assembling PNCs based on Fe_3_O_4_ NPs face the problem of aggregation or nonuniform distribution of the NPs, which leads to compromised functionality or even structural failure. To solve this problem, we use functionalized PDMS as the base material of the matrix, the structure of which is maintained by supramolecular interactions (Figure 1a). Films based on PDMS exhibit a favorable combination of a high coefficient of thermal expansion^26^ and a high compatibility with filler materials.^27^ Functionalized PDMS can work as a scaffold to maintain the integrity of the matrix and also generate a sufficiently large volume expansion when the nanofillers are activated by their specific stimuli. Fourier transform infrared (FTIR) spectroscopy is used to demonstrate the partial silylation of CNC. The infrared spectra of the MCP film indicate the presence of the methyl siloxy group at 1261 cm^–1^ and the hydroxyl group at 3350 cm^–1^ (Figure S1). This observation suggests that Si-CNC participates in constructing the supramolecular network of the matrix through hydrophobic interactions between silylated cellulose and PDMS, as well as hydrogen bonding with adjacent molecules.^28^ The presence of Si-CNC can lower the cross-linking density of the elastomeric network by forming interfacial interactions with PDMS, and it gives access to the possibilities of structural rearrangement of the matrix by exposing the H-bonding sites. With the dual role of photothermal conversion agents and magneto-responsive elements,^29^ the embedded Fe_3_O_4_ NPs are expected to form a thermal-conductive network and enable the dynamic cross-linked matrices to be reconfigured by heat, light, and magnetic fields.

We first studied the multiscale assembly of Fe_3_O_4_ NPs in the MCP films. Due to the richness of functional groups, Si-CNC can enhance the dispersibility of Fe_3_O_4_ NPs in PDMS. Scanning electron microscopy (SEM) and energy-dispersive spectroscopy (EDS) experiments are employed to investigate the agglomeration state and the distribution of the Fe_3_O_4_ NPs at the nanoscale, from which the results indicate that Fe_3_O_4_ NPs are homogeneously distributed without apparent aggregation (diameter smaller than 400 nm) in the Si-CNC/PDMS matrix (Figures S2a and S3). Despite the submicroscale homogeneity, SEM at a higher magnification displays nanoscale aggregates of Fe_3_O_4_ NPs with a diameter of up to 30 ± 5 nm (Figure S2b). This finding coincides with the observation of nanoaggregates and microscale agglomerates of Fe_3_O_4_ NPs in the mixture of Fe_3_O_4_ NPs, Si-CNC, and PDMS obtained from dynamic light scattering (DLS, Figure 1b). These multiscale Fe_3_O_4_ NP assemblies could arise either from the solution casting step or from multiple NPs being coated by the polymer matrix, with the latter one more plausible since the signal of Fe_3_O_4_ NP agglomerates is absent in the water dispersion of Fe_3_O_4_ NPs. We notice that the diameter detected by DLS is relatively larger than that of agglomerates detected by SEM. The likely reason is that the diameter measured from DLS is the sum of the Fe_3_O_4_ assemblies and the polymer shell. The X-ray diffraction (XRD) analysis of Fe_3_O_4_ NPs reveals diffraction peaks at 30.1°, 35.4°, 43.1°, 53.4, 57.0°, and 62.5°, which can be indexed to the (220), (311), (400), (422), (511), and (440) planes of the surface-centered cubic structure of magnetite according to the reference pattern (JCPDS No. 19-0629) (Figure 1c). We use the full width at half-maximum of the most prominent reflection at 2θ = 35.4° in order to calculate the crystallite size, which is 8 nm according to the Scherrer equation.^30^ The transmission electron microscopy (TEM) results suggest an average particle radius of 7.5 ± 0.5 nm (Figure 1d). Collectively, these results indicate the multiscale assemblies of Fe_3_O_4_ NPs, which form nanoscale aggregates and microscale agglomerates in the functionalized PDMS matrix.

In the next part, the thermal responsiveness of the MCP film is studied in terms of magnetic properties by vibrating sample magnetometry (VSM). The measurements are performed by sweeping the external magnetic field between −25 and 25 kOe at 25 °C (Figure S4). The MCP film and the Fe_3_O_4_ NP/PDMS film fabricated by the same procedure are tested. The two films differ significantly regarding the type of magnetic behavior and the saturation magnetization (Ms) values. The Fe_3_O_4_ NP/PDMS film exhibits superparamagnetism without magnetic hysteresis and an Ms of 3.62 emu·g^–1^ at 25 °C (comparable to values for similar materials; see refs (31−33)), indicating its vivid magneto-responsive properties and the absence of large-scale iron oxide agglomerates contributing to the magnetic behavior. In contrast, the MCP film shows a classical hysteresis and no more superparamagnetic behavior. These observations underline the complexity, which can arise when mixing the magnetite nanoparticles and polymer matrices, as previously reported.^34,35^ This absence of superparamagnetic behavior can relate to nanoclustering and aggregation of the Fe_3_O_4_ NPs due to attractive van der Waals forces between the particles. The Ms of the MCP film is 2.62 emu·g^–1^ at 25 °C, which is to some extent lower than that of the Fe_3_O_4_ NP/PDMS film. This result is deduced to arise from the antiagglomeration effect of Si-CNC and the diamagnetism of the polymer shell, which could shield part of the magnetism of Fe_3_O_4_ NPs. Collectively, the nanocomposite film still possesses sensitive magneto-responsive properties despite the shielding from the polymer shell.

### Mechanothermal Responses

The modulation of the thermal environment is presumed to be one of the major sources of impacting the viscoelastic properties of the magnetic PNCs. Therefore, we further examine the thermal-responsive viscoelasticity of the MCP film by DSC and temperature-controlled DMA, which probe the heat absorption/dissipation ability of the MCP film and the mechanoresponse of the MCP films subjected to periodic tensile stress as a function of temperature, respectively.^36^ DSC is performed from 0 to 130 °C with a step of 10 °C. From the DSC curve in Figure 1e, we infer that the transition point of the Si-CNC/PDMS matrix is 64 °C, and it increases to 74 °C when Fe_3_O_4_ NPs are incorporated. This small peak (not to be mistaken for a glass transition) corresponds to the melting transition of the composite film and indicates that the incorporation of Fe_3_O_4_ NPs slightly increases the melting point of the Si-CNC/PDMS matrix. Since PDMS remains stable in the temperature range of testing,^37^ the melting transition of the MCP film is mainly attributed to the filler system of Si-CNC and Fe_3_O_4_ NPs, and more specifically, the synergy between the Si-CNC molecules and the Fe_3_O_4_ NP agglomerates that disorganize the cross-linking of PDMS.

Moreover, the DMA tests are conducted from −20 to 110 °C at a frequency of 1 Hz in the elastic range of the MCP films. The elastic range of the MCP films is quantified by tensile tests at 25 °C. As evidenced by the strain–stress plots (Figure 1f), the maximum strength and elongation-at-break of the Fe_3_O_4_ NP/PDMS film are 0.89 MPa and 384%, which become 4.18 MPa and 213% after the incorporation of Si-CNC, respectively. The reinforcing effect of Si-CNC can result from replacing the relatively soft PDMS with stiffer cellulose crystalline. On the other hand, the addition of Fe_3_O_4_ NPs can reinforce the Si-CNC/PDMS film by enhancing the maximum strength. The reinforcing effect could arise from the retarded dynamics of the polymer segments, which are in close contact with the NPs, resulting in chain stiffening.^38,39^

When the temperature increases gradually from 20 to 70 °C, the continuous decrease of the storage modulus (Figure 1g) suggests the softening of the MCP film, with the storage modulus decreasing from *E′* = 0.84 to *E′* = 0.6 MPa and loss modulus increasing from *E″* = 0.18 to *E″* = 0.32 MPa, respectively. This could also be evidenced by the evolution of the loss factor (tan δ), which shows a relatively broad temperature range from 30 to 110 °C. A relatively slow increase in tan δ from 30 to 70 °C suggests enhanced molecular dynamic behavior of the polymeric matrix, potentially through the introduction of defects with the deaggregation of Fe_3_O_4_ NP assemblies and/or a higher degree of exposure of the H-bonding sites of Si-CNC. These physical cross-linking associations allow for an increased chain mobility of the components of the MCP film at an elevated temperature. Thus, the viscoelastic properties of the film change considerably, from which the softening of the polymer matrix may lead to the emergence of various thermal-assisted reconfiguration processes, such as healing, welding, remolding, and modular assembly. These reconfiguring possibilities are studied in the following section.

### Reconfiguration of the MCP Film through Thermal Mechanoresponses

**Figure 2:**
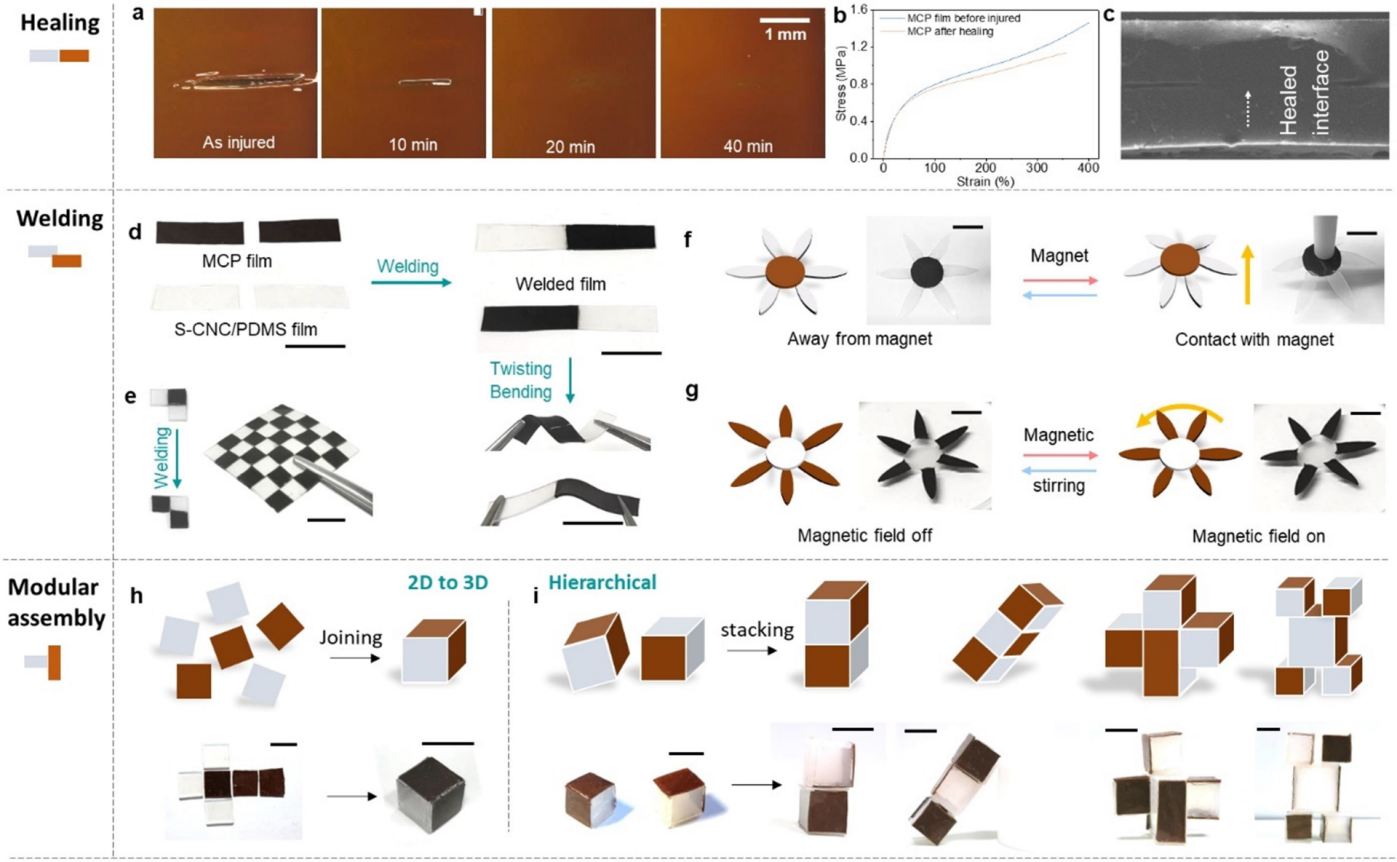
Thermal-assisted reconfiguration of the MCP films. (a) Optical microscopic observation of the thermal-induced healing of the MCP film with a scratch as a representative type of injury. (b) Comparison of the tensile strain–stress curves between a healed film and a noninjured MCP film. (c) SEM micrograph of the welded MCP films. The white arrow guides the eye to the welded interface. (d) Photographs showing the stretchability of the thermally welded films. An MCP film and a Si-CNC/PDMS film are used to demonstrate the heterogeneous welding ability. (e) Mosaic, square-shaped fabric obtained by heterogeneous welding. (f) Photographs showing the magnetic field adhesion of a thermally welded MCP flower to a magnetic bar (20 mT). The petals of the flower are composed of Si-CNC/PDMS films, and the core is the MCP film. (g) Photographs showing the magnetic-field-responsive rotation of a thermally welded MCP flower with the petals composed of MCP films and the core composed of the Si-CNC/PDMS film. A magnetic stirrer is employed as the rotating field (8 mT). (h) Schematics and photographs of the fabrication of a cube from two square-shaped MCP and four Si-CNC/PDMS 2D films. (i) Schematics and photographs for modular assembly of the cubes and cuboids in panel (h) into hierarchical 3D shapes. The scale bar is 10 mm.

Based on the DMA analysis, we deduced that the thermal mechanoresponses of the MCP film can provide the potential structural basis for various reconfiguration processes. To test this hypothesis, we investigated the healing function of the mechanical injuries of the MCP film in the first place. The healing phenomenon is tested by examining the morphological recovery process of the injured area of the MCP film by optical microscopy. A scratch is made in the MCP film, which gradually disappears by heating the film at 60 °C for 40 min (Figure 2a), verifying the heating-induced recovery of the injuries at the microscale. The reliability of the thermally assisted healing is also examined by tensile tests. The strain–stress curves of the healed sample are compared to those of the noninjured ones. The healed sample restores a fracture strain of 355.6%, achieving a healing efficiency of 89% when compared to the 399.5% fracture strain of the original sample (Figure 2b).

In addition to healing, the thermal mechanoresponses of the MCP films also invite the ability of remolding and welding. As shown in Figure S5, the cut pieces of an MCP film and a Si-CNC/PDMS film are mixed, molded at 70 °C for 30 min under a pressure of 5 MPa, and reprocessed into intact films with the shape of an owl and a butterfly, respectively. The remolding capability of the MCP film is due to reversible H-bonds, which can be broken/reformed upon the application of adequate thermal stimulus and, potentially, the structural reorganization of the polymer network that takes place within the PNCs. With the remolding capability, the MCP film can be prepared into complex 2D shapes.

Welding of the MCP film is achieved by three steps: overlapping two pieces of MCP films at an assigned region; heating the overlapped films at 60 °C for 30 min to enable the rearrangement of the PNCs; and pressing the overlapped films gently to make them well contacted. The interface between the two welded MCP films is nearly invisible from the cross-sectional SEM observation (Figure 2c). Despite welding two MCP films of the same chemical composition, we also weld the MCP film with the chemically different Si-CNC/PDMS film as a demonstration to construct multimaterial actuators (Figure 2d,e).

The heterojunctions in the welded samples can be visualized by the distinct colors of an MCP film (dark, with Fe_3_O_4_ NPs) and a Si-CNC/PDMS film (transparent, without Fe_3_O_4_ NPs). The strategy of heterogeneous welding not only provides a customizable, modular approach that integrates various material components with distinct functionalities but also allows for the construction of actuators with more complex shapes and patterns. The fusion of the nonmagnetic Si-CNC/PDMS film to the MCP film brings in more modulated, controllable actuation patterns through the hybrid structures. As a proof of concept, we fabricate two flower-shaped magnetic actuating devices through welding and test the anisotropic responses of these two devices under a magnetic field applied perpendicular to the film plane. In the first device, a round MCP film is used as a core and welded to six petals of Si-CNC/PDMS films (Figure 2f). Actuation experiments are performed using a magnetic stirrer retriever bar with a center magnetic field strength of 20 mT perpendicular to the operation plane. After welding, the flower’s core adheres to the magnet when it approaches from which the magnetic forces could sustain the gravity of the whole device (Movie S1). In the second device, a round Si-CNC/PDMS film is used as the core and welded to six petals of MCP films (Figure 2g). When being subjected to a rotating magnetic field with a strength of 8 mT, the whole device could flow in the anticlockwise direction at a speed of 3.2°·s^–1^ (Movie S2). These flower-shaped devices can potentially serve as sensors of an approaching magnetic field.

Different from the existing fabrication methods of magnetic PNCs that achieve actuation patterns based on the anisotropic assembly of magnetic nanoparticles with predesigned alignments, we demonstrate that the actuating devices based on the MCP film show the potential of programmable and customizable actuation functions through controlling the welding patterns. Thermal-assisted welding of the PNCs is also important for fabricating hybrid devices or repairing damaged devices.^40^

**Figure 3:**
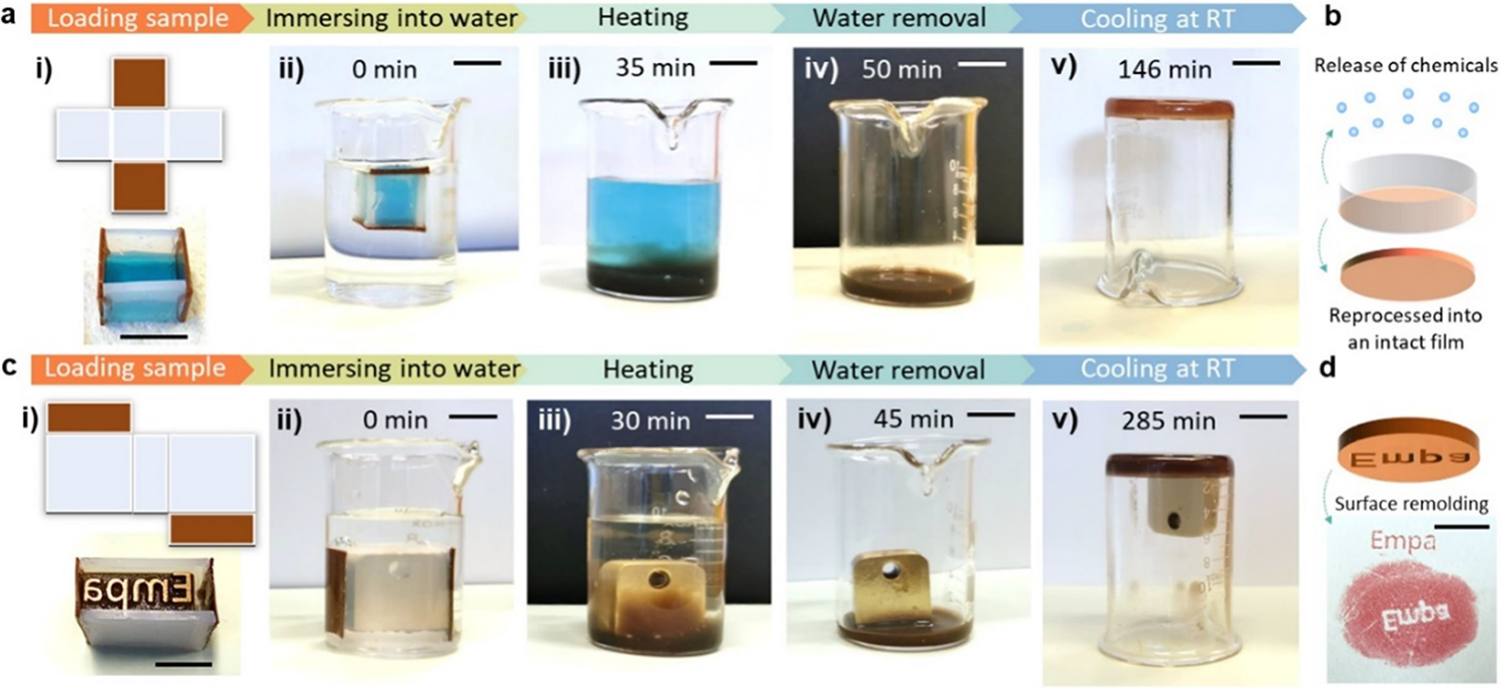
Sealing and releasing functions of the modularly assembled MCP containers. (a) Controlled release of water-soluble chemicals from an MCP container: (i) loading soluble ink; (ii) immersing the sealed container into DI H_2_O; (iii) heating at 70 °C; (iv) removing the aqueous medium; and (v) reforming the MCP film from the remaining components at room temperature. (b) Schematic illustration of the envisioned applications: releasing water-soluble chemicals and recycling of the container. (c) Controlled release of insoluble cargos from a MCP container: (i) loading a metal stamp; (ii) immersing the sealed cube into DI H_2_O; (iii) heating at 70 °C; (iv) removing the aqueous medium; and (v) the remaining MCP film components and the stamp are placed at room temperature. (d) Logo molded from the original stamp (top) and molded from the mirror-symmetric version of the reformed MCP film (bottom). The scale bar is 10 mm.

Furthermore, we assemble the 3D structures by “joining” the 2D MCP films into various 3D shapes, as well as by “stacking” the assembled 3D shapes into hierarchical structures. To better visualize the assembly pathways, the MCP films and the Si-CNC/PDMS films used for demonstrating the hierarchical assembly are produced at different manufacturing steps. First, the modular cubes are assembled from six pieces of the MCP films and the Si-CNC/PDMS films as the basic units (Figures 3h and S6). Second, these modular cubes are assembled into hierarchical 3D objects (Figure 3i, left) through heating, followed by stacking them through the heated surfaces of the Si-CNC/PDMS films. The detailed fabrication steps are described in Supplementary Note 1. As a proof-of-concept demonstration, we fabricated a “handbag” and a “snowboard” to show the potential of the 3D-assembled geometrical objects made from the MCP films for skiing activities (Figure S7), combining the strategies of welding and joining.

Following this route, we envision that diverse and hierarchical 3D shapes can be obtained by reconfiguring the units of 2D MCP films with simple initial shapes. By using the procedures including welding, joining, and stacking,^41^ the range of applications of the MCP films could be greatly broadened. Like the healing and remolding abilities, the modular assembly of 3D shapes is also obtained through the thermal mechanoresponses of the PNCs. However, the differences between the 3D assembly and healing/remolding are that (1) the units of the MCP films are joined by applying the thermal stimulus locally instead of universally and (2) the networks at the joint interfaces are heated at their melting temperature for a relatively shorter time compared to healing and remolding to avoid losing the structural stability of the units. Therefore, the different units can be stably fused, enabling repeated reconfiguration of the MCP films repeatedly.

### Controlled Releasing Functions Induced by Thermal-Assisted Reconfiguration

To demonstrate the multifunctionalities of the MCP films enabled by modular assembly, we use the MCP cubes as containers to encapsulate and release the liquid and solid objects through temperature control, respectively. The detailed experimental procedures are described in Supplementary Note 2. The sealing and releasing of the blue ink (Finecolor) from the MCP container indicate the potential of the MCP films as customizable and reusable containers for the controlled release of soluble chemicals (Figure 3a–d). To demonstrate the solid encapsulation functions of the MCP containers, a metal stamp with the logo “Empa” is used as the cargo, which is gently released from the softened container when heated to 70 °C, and the mirror-symmetric logo of the stamp is obtained after the film is cooled to room temperature (Figure 3e–f). These experiments indicate that, despite reconfiguring their shapes into the desired mold geometry, the MCP containers are also promising for transforming into adaptable devices with customizable shapes for flexible 3D fabrication. Moreover, the reshaped MCP containers can be recycled through a heating–cooling cycle.

It is noteworthy that thermal-induced softening of the MCP film might compromise the stability of the as-fabricated devices to a certain extent when being subjected to a temperature above its melting point. However, when the actuators or containers made from the MCP films are applied in a suitable temperature range and the long period use at excessively high temperatures is reduced, weight loss can be avoided.

### Microscale-Restructuring Investigation during the Injury–Healing Process

**Figure 4:**
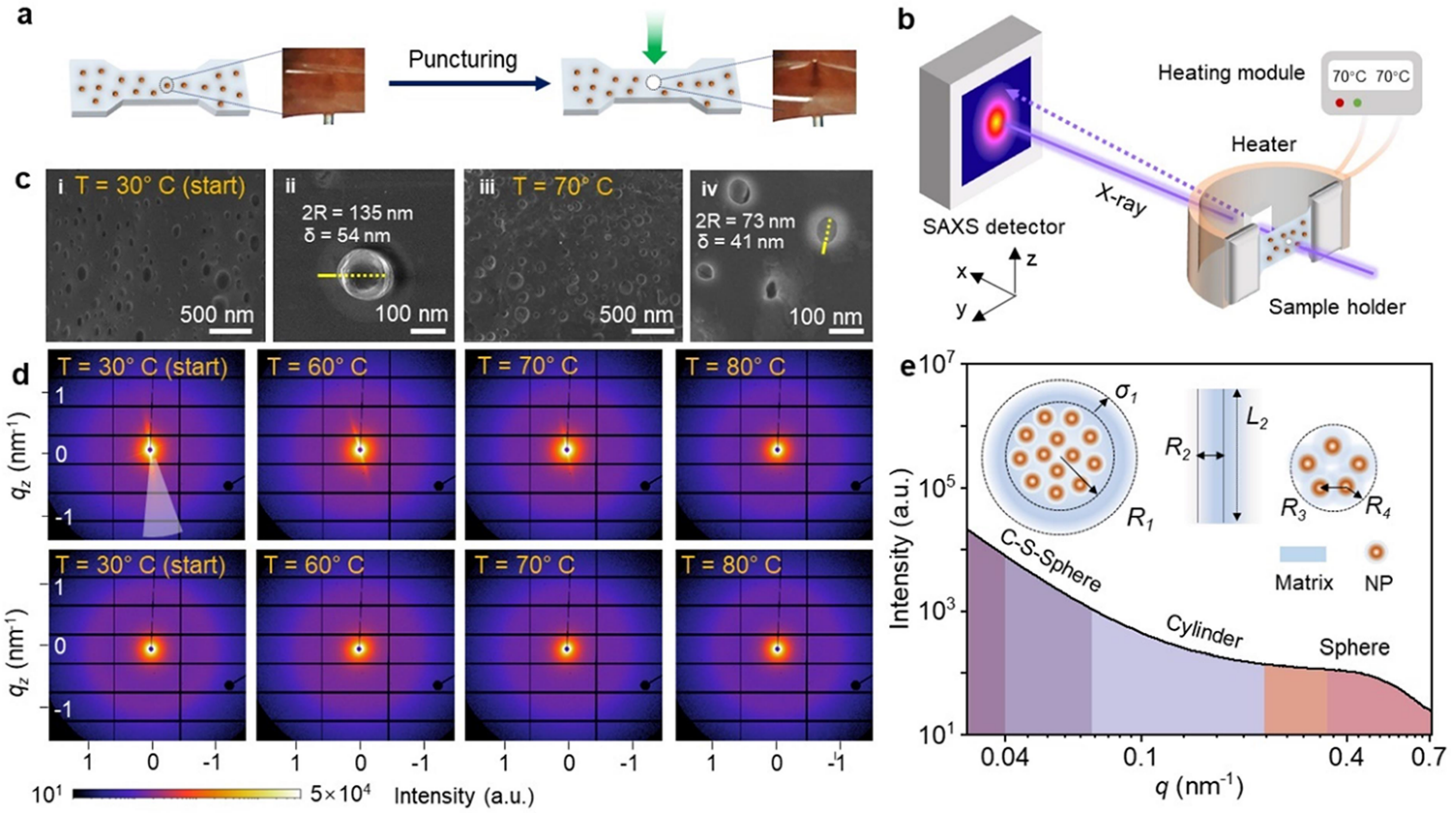
Temperature-controlled SAXS study of the MCP film. (a) Schematic and photographic illustration of the puncturing of the MCP film, which is intended to create an injured area. (b) Layout of the SAXS experiment. A heating module is used to control the temperature, which is varied to increase from 30 to 80 °C (heating process) with a step of 10 °C and decrease to 30 °C (cooling process) again. A stretchable sample stage is used to keep the MCP film straight. (c) Cross-sectional SEM micrographs showing submicroscale Fe_3_O_4_ NP agglomerates capsulated by the Si-CNC/PDMS matrix before (left, (i) and (ii)) and after (right (iii) and (iv)) heating at 70 °C for 20 min. The core–shell structure is clearly visible before heating (ii), but the boundary between the core–shell gradually disappears after the heating process (iv). (d) Representative 2D SAXS images of the MCP film at the center of the injured area (top panel) and noninjured area (bottom panel) during the heating treatment. (e) Structural model with three geometrical objects, which are used to fit the SAXS data, including a core–shell sphere to represent the Fe_3_O_4_ NP agglomerates capsulated by the polymer matrix at the submicroscale, a cylinder to fit the polymer matrix, and a sphere to fit the Fe_3_O_4_ NP clusters at the nanoscale.

To investigate the potential structural mechanisms for thermal mechanoresponses and the subsequent thermally assisted reconfiguring properties of the MCP films, in situ SAXS experiments are performed under temperature control. SAXS is suitable for the in situ investigation of the thermal-induced structural responses of the MCP film for three reasons: (1) the relatively broad melting transition temperature range (70 ± 5 °C, Figure 1e) makes it, gratifyingly, possible to study the real-time structural evolution of the film by 2D screening of the injured area without inducing rapid melting of the material; (2) the structural organization of the fillers and the polymer matrix can be determined independently in the same sample due to their different X-ray scattering length densities (see the Experimental Section); (3) SAXS provides a suitable detection range for investigating the nano- or microscale structures, of which the rearrangement is supposed to contribute to the thermal-induced healing of the MCP film. We study the injury–healing process as a simplified system for the thermal-induced structural responses of the MCP films. Due to the highly elastic property, a punctured area (0.5 × 0.5 mm^2^) is applied to the MCP film to represent the possible damages (Figure 4a).

The in situ SAXS measurements for studying the heating-induced restructuring of the MCP film are conducted at the P03 beamline of PETRA III (Deutsches Elektronen-Synchrotron DESY).^42^ We perform a stepwise heating procedure in the temperature range from 30 to 80 °C and then return directly to 30 °C. A heating module is mounted onto a specialized sample holder compatible with X-ray scattering geometry (Figures 4b and S9) to create a heat flow surrounding the MCP film.^43^ SAXS data are collected before, during, and after the heating procedure, respectively. The MCP film is scanned in both the *q*_*z*_ and *q*_*y*_ directions with a step width of 0.04 mm and 9 scan points in each direction to obtain a 2D topography map of the injured area. The 2D scan positions are written as “*yrz*”, where “*y*” and “*z*” represent the scan position in the *q*_*y*_ and *q*_*z*_ directions, respectively (Figure S10). The 2D scan takes 20 min for each single temperature step, which is sufficient for us to compare the changes in scattering patterns at the same scan position during the heating procedure. The cross-sectional micrographs of the MCP film before heating show a smooth and uniform morphology of the matrix and the core–shell structure of the microscale Fe_3_O_4_ NP assemblies (Figure 4c, i and ii). However, the boundaries of the core–shell spheres become blurred when the MCP film is heated to 70 °C (Figure 4c, iii and iv).

The 2D SAXS data of the injured film area (Figure 4d, top panel) display a highly anisotropic pattern compared to the noninjured parts (Figure 4d, bottom panel). The fast deformation at the injured area causes irreversible damage and restricts the reconstruction of the filler network. While at the adjacent areas near the puncturing site, the supramolecular interactions in the MCP film destruct and reconstruct simultaneously, causing the lack of the nano- or microscale structures induced by the injury. However, we still observe the sharp difference as the yellow-colored streak between the center and the adjacent area of the puncturing site from the SAXS data at the starting temperature (30 °C, Figure 4d, left). In the range of 0.03 < *q* < 0.1 nm^–1^, a strong anisotropic scattering pattern is observed at the scan position of *3r5*, which might be due to the distorted hierarchy of the Fe_3_O_4_ NP assemblies. The scan position *3r5* is identified as the center of injury.

When the temperature is increased from 30 to 60 °C, this scattering pattern gradually evolves from an elliptical to a circular shape. The anisotropic scattering pattern diminishes at 70 °C, and the corresponding anisotropic peak becomes flatter. At 80 °C, more fluctuations are observed in the scattering curves at all scan positions. A shallow peak evolves and becomes increasingly pronounced in the range of 0.33 < *q* < 0.72 nm^–1^. When the MCP film cools down to 30 °C, the SAXS pattern at *3r5* can almost restore to a state similar to that of the noninjured area. This may signify the homogenization of the injury-induced and noninjured structures at the fractured interfaces. To evaluate the dynamic evolution of these structures, the 2D SAXS data are azimuthally integrated to obtain the 1D SAXS profiles for further analysis (Figure S11).

### Modeling the Heating-Induced Structural Reorganization

The 1D SAXS profile of the MCP film can be divided into three *q* regions, to which three geometrical objects are selected and used for the structural model of SAXS fits according to the cross-sectional morphology of the MCP film (Figure 4e). We identified the hierarchy of Fe_3_O_4_ NP assemblies at different length scales by SEM performed at different resolutions, including the relatively larger spherical structures with a protective shell and the relatively smaller Fe_3_O_4_ NP clusters, respectively (Figures 4c and S2b). Due to this hierarchical assembly, we consider the MCP film as a system with three geometrical objects: Fe_3_O_4_ NP agglomerates capsulated by the polymer matrix through interfacial interactions, Fe_3_O_4_ NP clusters through interparticle interactions, and the remaining Si-CNC/PDMS matrix via a supramolecular network.^4^ In other words, the particle–polymer, interparticle, and interpolymer interactions are considered in the model to be applied and fitted.

The low *q* region (0.031 < *q* < 0.072 nm^–1^) reflects the uniformly distributed and micron-sized agglomerates of Fe_3_O_4_ NPs, which might be caused by the fabrication procedure. These agglomerates are spaced by polymer segments, which can be strongly immobilized to form a shell layer near the Fe_3_O_4_ NP agglomerates. To account for the presence of the polymer shell, a core–shell sphere is used (Figure 4e, left). The high *q* range (*q* > 0.23 nm^–1^) reflects the Fe_3_O_4_ NP clusters at the nanoscale. Superparamagnetic NPs normally form nanostructures into submicron structures with multiparticle and isotropic assemblies due to magnetic interactions.^5^ These structures can be building blocks for larger-scale assemblies of the core–shell spheres. A sphere is used to represent the size along with a hard-sphere structure factor to describe the shape of small Fe_3_O_4_ NP clusters, which are related to the magnetic interactions between neighboring NPs (Figure 4e, right). A cylinder that represents the Si-CNC/PDMS matrix is used to fit in the mid *q* range (0.072 < *q* < 0.23 nm^–1^). The cylinder is composed of the rigid crystallinity of Si-CNC and the soft network of PDMS. Analyzing the mid *q* region with this object reduces the complexity of the soft polymer network with a relatively irregular shape (Figure 4e, middle).

**Figure 5:**
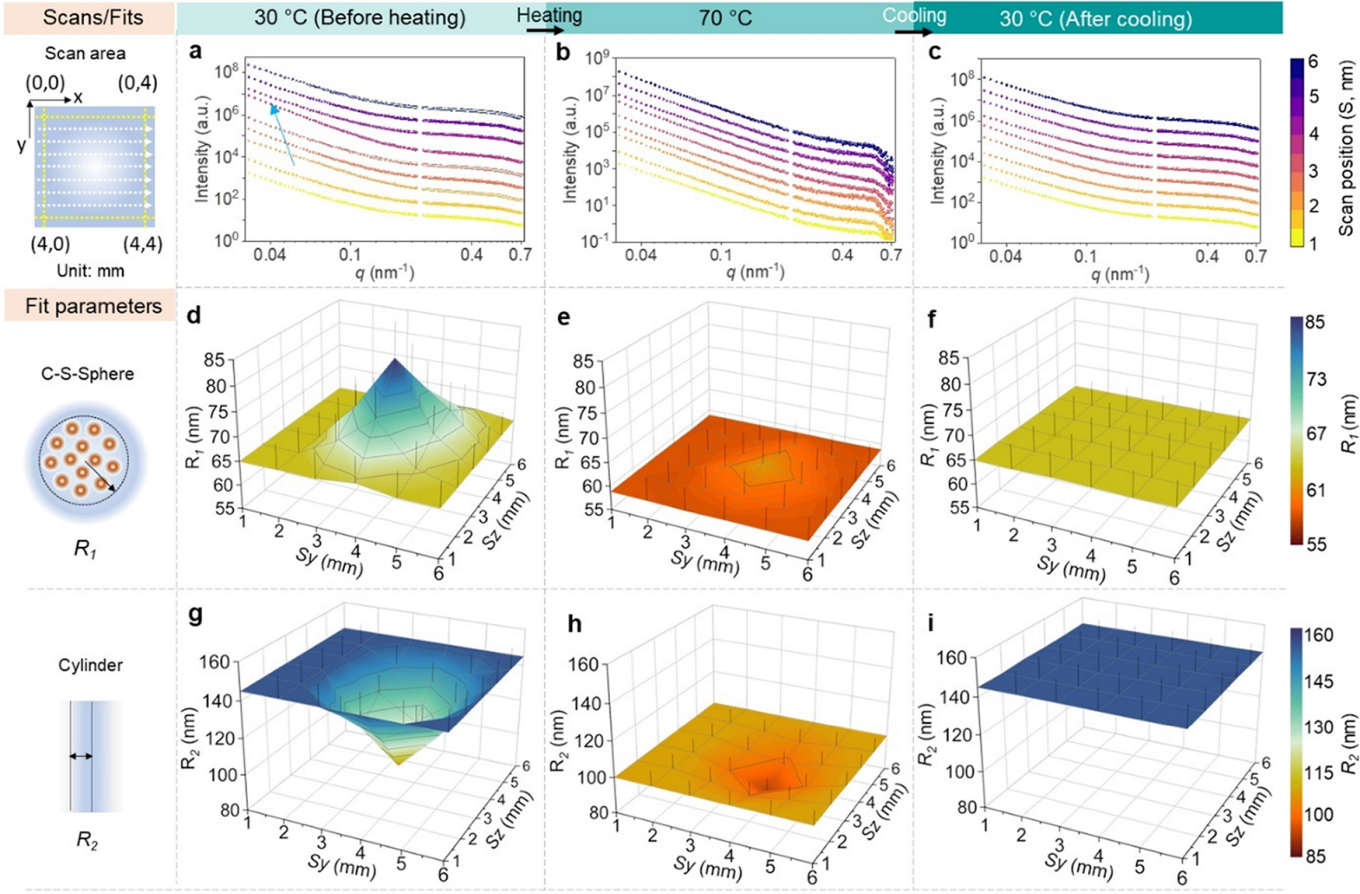
SAXS analysis of the thermal-induced structural reorganization of the MCP film. (a–c) Comparison of the 1D integrated SAXS data collected at 30 °C (heating, a), 70 °C (b), and 30 °C (cooling, c). The 1D integration data during the entire heating treatment are shown in Figure S11. (d–i) Representative fit parameters across the injured area, including the radii of the core–shell sphere (*R*_1_) and radii of the cylinder (*R*_2_) at 30 °C (heating, d, and g), 70 °C (e, h), and 30 °C (cooling, f and i), respectively. The other fit parameters can be found in Figure S13–S16.

The use of SAXS fit models helps in the interpretation of the evolution of nano- and microstructures of the MCP film during the heating procedure over an expansive length scale, which, in turn, correlates with a broad range in real space. For each fit, the radii of individual Fe_3_O_4_ NPs and polydispersity (size distribution) are kept constant. The radii of Fe_3_O_4_ NPs correspond to the value gathered from TEM (Figure 1d), and a Gaussian distribution is introduced to account for the polydispersity of the radii and length of the polymer matrix.^44^ The one-dimensional integration curves of the MCP film are shown in Figures 5a–c and S11, including the best fits. An example of a typical least-squares fit report is displayed in Figure S12. The evolution of the most important parameters of the MCP film during the heating procedure is plotted in Figure 5, including the radii of the core–shell sphere (d–f) and cylinder (g–i).

At 30 °C, a decreased intensity in the low *q* range from the periphery (*3r1*) to the center of injury (*3r5*) is observed, which suggests that the large Fe_3_O_4_ NP agglomerates move closer with an increased radius of the core from (*R*_1_ = 65.0 ± 5.2 nm) to (*R*_1_ = 84.0 ± 6.7 nm). The changes can hardly be observed in the sphere since the rearrangement of the large Fe_3_O_4_ NP agglomerates may result in insignificant statistical changes in the size of small clusters (*R*_3_ = 6.0 ± 0.5 nm). Also, we observe reduced radii and lengths of the cylinder from (*R*_2_ = 145 ± 11.6 nm, *L*_2_ = 310 ± 15.5 nm) to (*R*_2_ = 117 ± 9.3 nm, *L*_2_ = 283 ± 14.1 nm) as the scan position moves from *3r1* to *3r5* (Figures 5g–i and S13). The decrease in size parameters of the cylinder may contribute to the rearrangement of the initially adjacent Fe_3_O_4_ NP agglomerates, the debonding of the particle–polymer, and the ensuing fragmentation of the polymer matrix. The fit parameters remain unchanged when the temperature increases from 30 to 40 °C, start to change as the temperature increases from 40 to 50 °C, and become increasingly homogenized from 60 to 80 °C. The homogeneity is preserved during the cooling process, and the fit parameters return to their initial values after cooling back to 30 °C. The structural evolution starts at a lower temperature than that detected by DSC, likely because the microstructural changes may not be immediately transformed into macroscopic properties (Table S1). We analyze the fit parameters of the curves collected at 30, 60, and 70 °C, respectively, which are identified as three representative heating steps of the microstructural evolution.

Analysis of the low *q* region provides distinct insight into the evolution of large Fe_3_O_4_ NP agglomerates. The micrometer-sized spheres fit from SAXS corroborate the observation from SEM, which is (65 ± 10 nm) and (45 ± 8 nm) at the center of injury before and after the heating procedure, respectively. For the core–shell sphere, as the temperature increases from 30 to 60 °C, the radii display a strong decrease from (*R*_1_ = 84 ± 7 nm) to (*R*_1_ = 70 ± 6 nm) at the center (*3r5*), and a slight decrease from (*R*_1_ = 65 ± 5 nm) to (*R*_1_ = 60 ± 5 nm) at the periphery (*3r1*) of the injury, respectively (Figures 5d–f and S13a–c). The decreased radii qualitatively indicate an increase in the quantities of isolated small Fe_3_O_4_ NP clusters. In other words, the Fe_3_O_4_ NP agglomerates disassemble and reassemble in this temperature range. The changes are more prominent along the thickness of the shell than at the core, which reduced from (σ_1_ = 60 ± 5 nm) to (σ_1_ = 35 ± 4 nm) at the center, and from (σ_1_ = 46 ± 4 nm) to (σ_1_ = 29 ± 4 nm) at the periphery of the injury in the temperature range from 30 to 60 °C, respectively (Figure S14a–c). The decrease at the center is faster than that at the periphery. The decreased thickness indicates that one Fe_3_O_4_ NP is bound with a reduced amount of the polymer matrix on average since a larger quantity of small Fe_3_O_4_ NP clusters is evolving.

From 60 to 80 °C, the difference in the core–shell spherical structures narrows between the center and the periphery of the injured area. For example, at 70 °C, the radii are (*R*_1_ = 63 ± 5 nm) and (*R*_1_ = 59 ± 5 nm) and the thicknesses are (σ_1_ = 29 ± 3 nm) and (σ_1_ = 26 ± 2 nm) at *3r5* and *3r1* scan positions, respectively (Figures S13d and S14d). Essentially, this decrease indicates that the filler/matrix interface becomes smoother when it is heated near the melting temperature of the MCP film, which is consistent with our speculation of the formation of smaller Fe_3_O_4_ NP assemblies. After cooling to 30 °C, the size parameters of the core–shell sphere homogenize and return to a similar state before the injury (Figures S13f and S14f). The increase in the radii of the core–shell sphere after cooling signifies that the Fe_3_O_4_ NPs reagglomerate below the melting temperature due to the viscoelasticity of the matrix and the interparticle interactions.

Scattering profiles within the mid *q* range correspond to the length scale occupied by the cylindrical Si-CNC/PDMS matrix. They represent the spaces between the adjacent Fe_3_O_4_ NP agglomerates. The cylinder shows a decrease in radii (Figures 5g–i and S15) and length (Figure S16) during heating at both the center and periphery of the injured area. At the scan position of *3r1*, the radius decreases from (*R*_2_ = 145 ± 12 nm) to (*R*_2_ = 124 ± 10 nm), and the length decreases from (*L*_2_ = 310 ± 16 nm) to (*L*_2_ = 249 ± 13 nm) when the temperature increases from 30 to 60 °C (Figures S15a–c and S16a–c). The injury center (*3r5*) achieves a slower rate of changes due to the larger space, in which the Fe_3_O_4_ NP agglomerates are embedded, with the radii decreasing from (*R*_2_ = 100 ± 8 nm) to (*R*_2_ = 92 ± 7 nm), and the length decreasing from (*L*_2_ = 250 ± 13 nm) to (*L*_2_ = 225 ± 12 nm). The structural parameters of the cylinder become uniformly distributed throughout the sample at 50 °C and continue to decrease from 60 to 80 °C due to the evolution of small Fe_3_O_4_ NP assemblies. For example, the radius decreases from (*R*_2_ = 92 ± 7 nm) to (*R*_2_ = 85 ± 7 nm), and the length decreases from (*L*_2_ = 225 ± 12 nm) to (*L*_2_ = 197 ± 10 nm) as the temperature increases from 60 to 80 °C at *3r5* (Figures S15d,e and S16d,e). The evolution of the cylinder is a complex process involving the reorganization of the Fe_3_O_4_ NP agglomerates and the softening of the Si-CNC/PDMS matrix.^45,46^ The onset of structural changes in the cylinder coincides with that of the core–shell sphere, which, in turn, is influenced by the fluidity of the matrix. Upon cooling to 30 °C, the structural parameters reach the same level as the starting point (Figures S15f and S16f). These changes imply that the thermodynamic equilibrium of the polymer matrix is reconfigured at an elevated temperature, which may account for the thermoresponsiveness of the MCP film.

The data in the high *q* region depict the differences in not only the size of the Fe_3_O_4_ NP clusters but also their correlations. For the heating procedure used here, the inner structure of individual Fe_3_O_4_ NPs, as well as the interparticle distance, is stable without clustering or degradation of the NPs.^47^ Therefore, the radii of the hard sphere and sphere are fixed at (*R*_3_ = 4.0 ± 0.3 nm) and (*R*_4_ = 7.3 ± 0.5 nm), respectively. The similar values between the radii of the sphere and the hard sphere indicate the close packing of the Fe_3_O_4_ NPs in the clusters. Furthermore, by keeping the polydispersity, particle size, and particle correlations constant for each fit, the scale of the sphere also correlates proportionally to the volume fraction of the Fe_3_O_4_ NP clusters. The intensity at the high *q* region initially remains constant from 30 to 50 °C, increases sharply from 60 to 80 °C, and decreases back to its original intensity after cooling to 30 °C (Figure S11). The increase in intensity from 60 to 80 °C indicates the development of newly formed Fe_3_O_4_ NP clusters, which originate from the reorganization of Fe_3_O_4_ NP assemblies at the higher level of the hierarchy.

**Figure 6:**
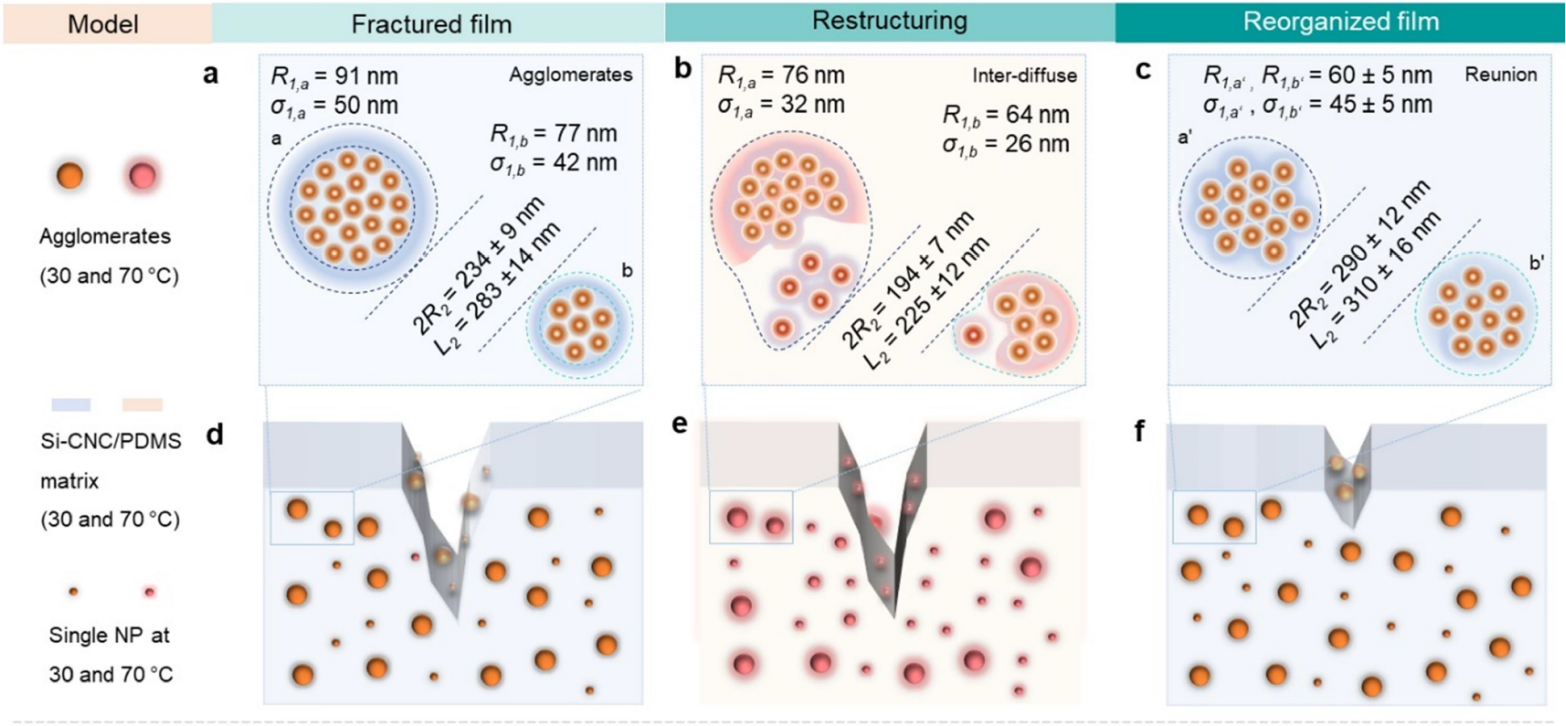
Structural model for heating-induced structural reorganization of the MCP film. Schematic illustration of the heating-induced structural rearrangement of the MCP film deduced from SAXS fits. The fit parameters marked in panels (a–c) are representative structural parameters of the core–shell spheres and cylinders at the center of injury.

We attribute the healing capability of the MCP film to the synergistic interactions of the supramolecular network, which enable sufficient chain mobility for healing while maintaining the integrity of the network for stretching. These interactions include covalent cross-linking of PDMS (critical for elasticity), particle–polymer interface interactions (important for heat transfer), and hydrogen bonding (essential for healing). As Fe_3_O_4_ NPs interact with the polymer chains mostly by physical cross-linking, these particle–polymer interactions cannot be reconfigured at room temperature as the polymer chains are kinetically trapped around Fe_3_O_4_ NPs. Hence, no healing is observed below the melting temperature of the MCP film (Figure 6a,d). However, when the temperature approaches the melting point of the film, the chain mobility of Si-CNC increases via the heat transfer from the Fe_3_O_4_ NP assemblies, dynamic H-bonding regions at the particle–polymer interfaces, and softening of the Si-CNC/PDMS matrix. Careful control of the heating condition can avoid melting the film and protect it against losing the structural integrity of the polymer network.

Although the chain mobility of Si-CNC at the fractured interfaces might decrease to some extent due to the loss of water, the enhanced thermal expansion of the PDMS matrix could accelerate the narrowing of microscopic cracks and bring the −OH groups into close contact. These combined actions reorganize the Fe_3_O_4_ NP assemblies^48^ and reconnect the H-bonds at the two broken surfaces (Figure 6b,e). When the temperature decreases, the Fe_3_O_4_ NP assemblies are immobilized in the solidified matrix, yielding a responsive material (Figure 6c,f). Compared to the materials healed at room temperature, the thermal-induced structural rearrangement of the MCP film enables the healing ability in a controllable manner, which can refrain from stickiness, self-adhesion, and low modulus that may limit the work output of as-fabricated devices.^22,49^

### Photothermal Conversion Ability

**Figure 7:**
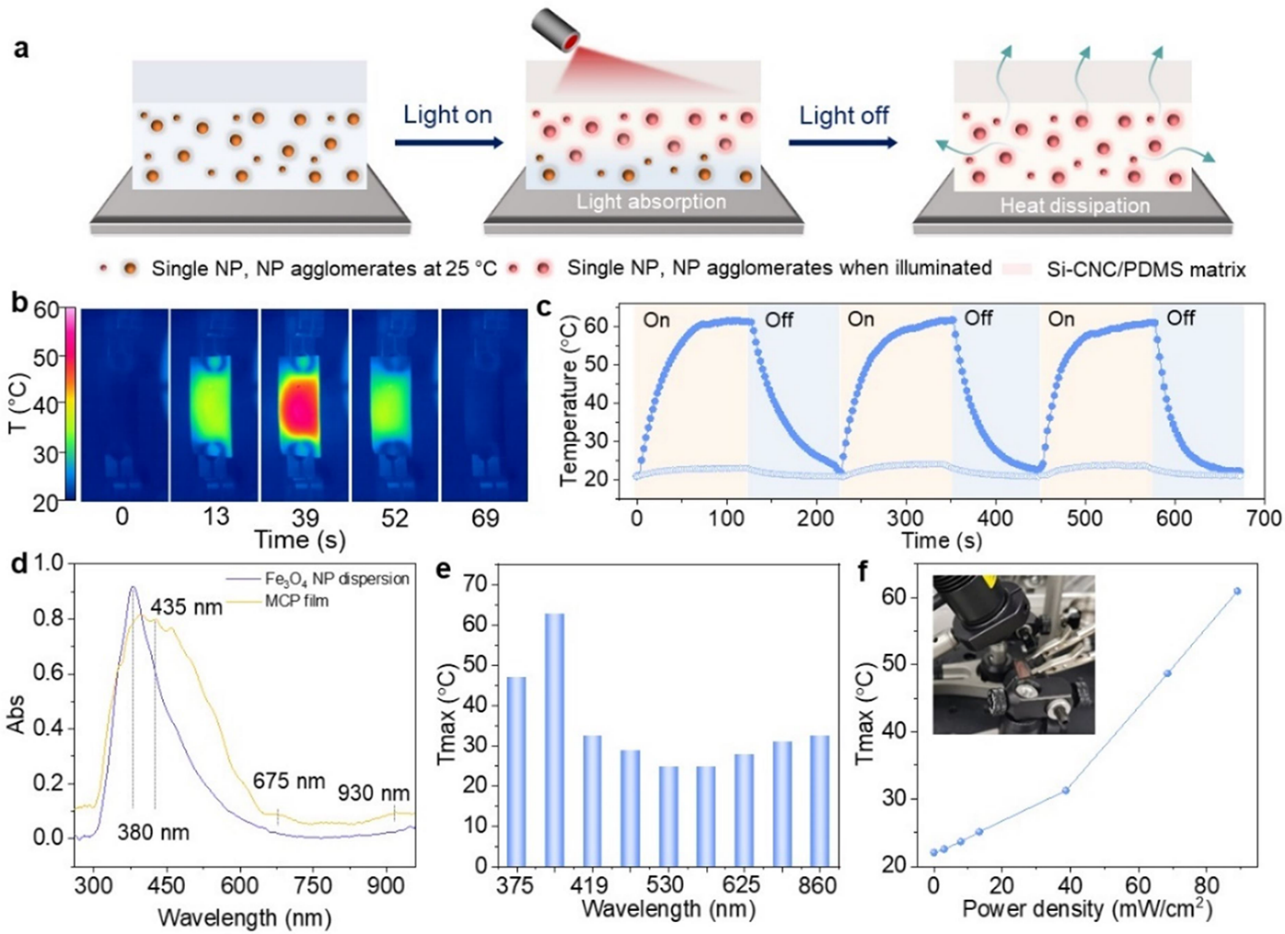
Spatiotemporally controlled photothermal conversion of the MCP film. (a) Schematic illustration of the photothermal conversion function of the MCP film. (b) Temperature maps of the MCP film recorded by an infrared camera. The temperature recording starts when the infrared (IR) LED is switched on and ends when the temperature returns to 25 °C. The LED is switched off when an equilibrium temperature is obtained (λ = 780 nm, power density = 28 mW·cm^–2^). (c) Time-dependent temperature variation of the MCP film in three IR on–off cycles. (d) UV/vis absorption spectrum of the aqueous dispersion of Fe_3_O_4_ NPs and the MCP film. (e) Temperature of the MCP film under the illumination of LEDs at different wavelengths and the power density of 89 mW·cm^–2^. (f) Temperature of the MCP film under the illumination of an IR LED (λ = 780 nm) at different power densities. Inset: Photograph of the experimental setup for temperature recording with an infrared camera.

The nano- to submicron structures of the Fe_3_O_4_ NP assemblies are beneficial for the photothermal properties of the MCP film because of the light trapping via multiple internal reflections. Photoillumination using mild light sources can generate localized heat in the Fe_3_O_4_ assemblies without destroying the structural integrity of the polymer matrix (Figure 7a). To study the photoresponsiveness of the MCP film, we first investigate the UV/vis absorption spectra of the water dispersion of Fe_3_O_4_ NPs since Si-CNC/PDMS can be regarded as transparent, and their absorption is negligible. The absorption peak of Fe_3_O_4_ NPs is relatively sharp at 380 nm and nearly flat in the IR range (Figure 7d). However, the Fe_3_O_4_ NP assemblies in the polymer matrix at various agglomeration degrees lead to a broader absorption range, even to the infrared (IR) range of the spectrum.^50^ Besides, IR light has a stronger photothermal effect and deeper penetration than short-wavelength light and is widely used in photoheating. Therefore, we use an IR LED (λ = 780 nm) as the light source for investigating the photothermal responsiveness of the MCP film.

The internally embedded Fe_3_O_4_ NP assemblies dispersed in the polymer matrix can work as photothermal converters and contribute to the photothermal conversion function of the film. The temperature field induced by light illumination and the photothermal effect of the Fe_3_O_4_ NPs is recorded by an infrared camera (Figure 7b,c). The schematic diagram of the testing setup is illustrated in Figure S17. The time-dependent temperature variations suggest that different heating rates are achieved when the films are exposed to a light source at different wavelengths (Figures 7e and S18) and power densities (Figure 7f), which are generally in accordance with the trend that we observe in the UV/vis absorption spectra. Under IR LED irradiation at 89 mW·cm^–2^, the MCP film can be heated to 65 °C in 53 s (Movie S3). When the LED is switched off, the irradiated region cools down to 21 °C in 40 s. In addition, the attained maximum temperature is proportional to the input power (Figure 7f).

Moreover, the 2D temperature distribution maps of the film (Figure 6b) suggest that the center of the film displays a higher temperature than the periphery under the illumination of the IR LED. This phenomenon could be due to the coupling of Fe_3_O_4_ NPs and the connectivity of the Fe_3_O_4_ assemblies in the PNCs. These results suggest that the MCP film can be actuated by light in a spatiotemporally tunable manner. The photothermal conversion function of the MCP film is important for its application as an actuator since the multifunctional platform of the MCP film may achieve more sophisticated sensing and actuation capabilities when combined with other heat-irresponsive materials.

### Outlook

**Figure 8:**
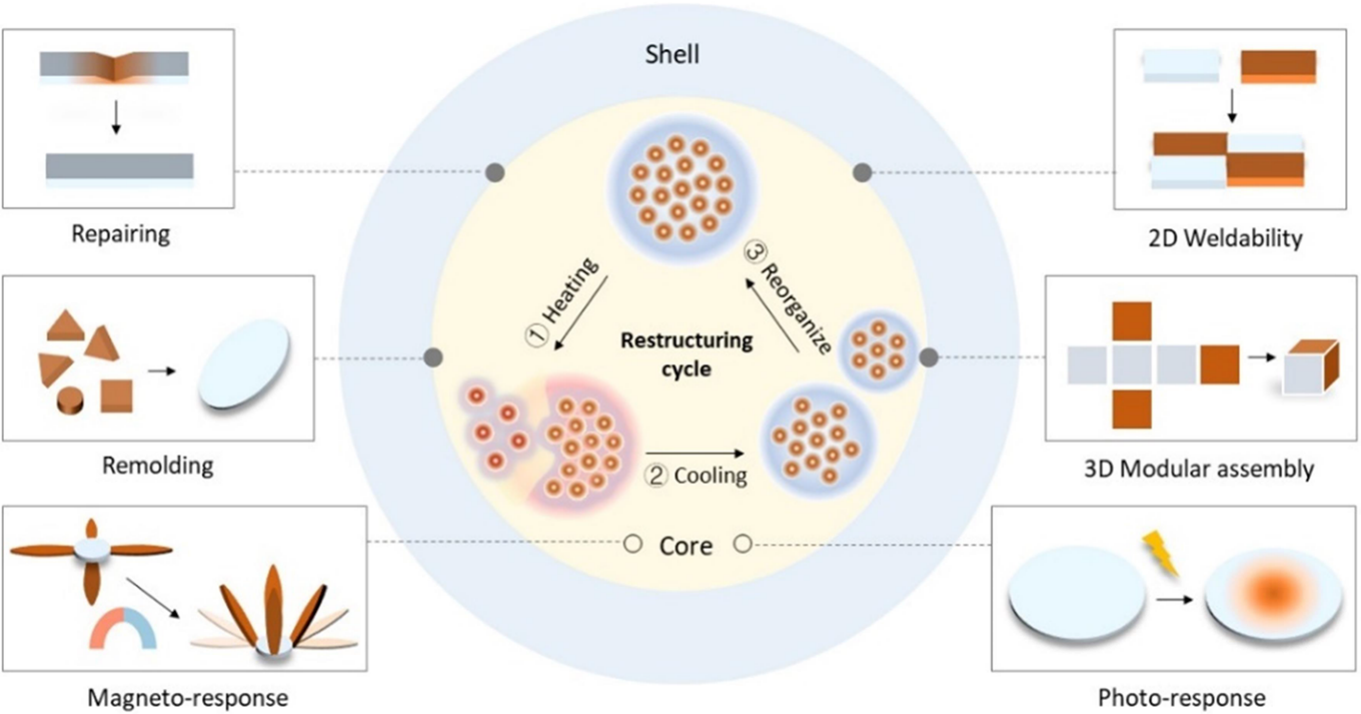
Thermally reconfiguring pathways of the MCP film through structural reorganization of the PNCs and the stimuli-responsive properties of the MCP film through the Fe_3_O_4_ NPs.

By leveraging the dynamic H-bonds in the polymer network, 3D shapes can be modularly assembled from the 2D MCP films, which are thermally treated beforehand to obtain assemblies with seamless joints. During the welding, joining, and stacking procedures, both the MCP films and the Si-CNC/PDMS films remain solid and can preserve their mechanical stability. These modular assembly techniques give rise to various advantages of the MCP films: (1) high manufacturing scalability because of the feasible conversion of 2D films into 3D structures; (2) easy incorporation of multimaterial components and custom-designed heterogeneous structures; (3) adaptation to different environments and tasks due to that the modularly assembled devices are collections of a series of autonomous elastomeric units based on the MCP films, which are connected to act as a whole entity; and (4) 3D structures assembled from hollow MCP cubes can be used as sealable containers for the encapsulation and release of liquid or solid objects in a thermally controllable manner (Figure 8). We envision that the structurally reconfigurable MCP films could be used to design adaptive smart packaging materials and 3D reconfigurable actuators.

## Conclusions

In conclusion, we designed a PNC with magnetite nanoparticles as fillers and a dynamic polymer network as the encapsulating matrix. The nano- to microstructures of the PNCs, including the organization of Fe_3_O_4_ NPs and the polymeric shells, can be reconfigured by the heating-induced solid–liquid phase transition. Through the combined in situ analytical approaches, we elucidate the impact of heating on the nano- to microscale structure and the viscoelasticity of the composite film. The presence of an encapsulating polymer shell has an essential role in reorganizing the Fe_3_O_4_ NP assemblies, which in turn leads to disruption and reconstruction of the polymer network of the PNCs. Thanks to the capability of thermal-induced softening and reorganization, we also demonstrate the possibilities of diverse actuating functions through welding heterogeneous films, healing of microscale injuries, and modular assembly of hierarchical 3D shapes out of the MCP films. Furthermore, we detect the responses of the MCP films to photo- and magneto stimuli. It should be noted that there are still versatile ways to combine the functions mentioned above in this dynamic system. With the incorporation of a more advanced design, such as laminating the film of the magnetic PNC onto a hygroscopic PNC film, we can anticipate a multiresponsive soft actuator with more customized architectures and functionalities. Therefore, the thermal-assisted structural rearrangements at the nano- to microscale represent a promising strategy for reconfiguring the stimuli-responsive PNCs at the macroscale. Based on these achievements, we anticipate that this stimuli-responsive PNC holds great promise in diverse applications wherever programmable structural rearrangement will be necessary, such as in smart packaging materials and soft robots.

## Experimental Section

### Chemicals and Materials

30 wt % aqueous dispersion of oleic-acid-stabilized Fe_3_O_4_ NPs with a diameter of 8 ± 3 nm was kindly provided by PlasmaChem GmbH, Germany. The concentration of Fe_3_O_4_ NPs in the dispersion was 1.3 mmol·L^–1^. Polydimethylsiloxane prepolymer (Sylgard 184A) and curing agent (Sylgard 184B) were purchased from Credimex AG, Switzerland. Si-CNC was kindly provided by Professor Kai Zhang and co-workers at the University of Göttingen. Si-CNC was synthesized by a method described in the literature.^28^ The water dispersion of Si-CNC was freeze-dried and redispersed in toluene (100 mg·mL^–1^) for melt-blending with PDMS.

### Film Preparation

PDMS prepolymer and curing agent were mixed with Si-CNC toluene dispersion and Fe_3_O_4_ NP water dispersion at a volume ratio of 2:20:1 by melt-blending. The mixture was stirred vigorously until a completely uniform mixture formed. The resulting mixture was poured into a precleaned glass Petri dish before degassing at 25 °C for 30 min. Finally, the mixture was cured at 25 °C for 2 days.

### Fourier Transform Infrared Spectroscopy

The FTIR spectra of the MCP film were recorded under ambient conditions (*T* = 25 °C, RH = 30%). The reflectance infrared spectra were collected by a Bruker Vector 22 spectrometer with a diamond cell attenuated total reflection accessory module in the range of 800–4000 cm^–1^. The FTIR scan took 40 s. Three specimens were tested, and one representative spectrum was used for analysis.

### Temperature-Controlled Dynamic Mechanical Analysis

Temperature-responsive viscoelastic behavior of the MCP film was performed on a DMA Q800 device (TA Instruments, New Castle, Delaware, United States). After the sample was placed under tension between a fixed and a moveable clamp, the heating chamber was closed to obtain the desired temperature range. The temperature was increased stepwise with a waiting time of 10 min for equilibration of the samples between the individual steps, and each sample was scanned at a given temperature range from 0 to 120 °C during a period of 120 min. All tests were conducted at a frequency of 1 Hz and a linear range of 0.5% strain. For each sample group, three separate measurements were taken to generate an average DMA response. The data were acquired and analyzed by Orchestrator v7.2.0.4 software.

### Tensile Test

The mechanical property of the MCP film was examined by a Zwick Z005 testing machine. Samples for mechanical stretching were cut with a size of 40 × 8 mm (length × width). Three stripes of each sample were tested under ambient conditions (*T* = 25 °C, RH = 30%). The top clamp was attached to a 50 N load cell. The stretching rate was 10 mm·min^–1^. The average thickness of the MCP film was 300 ± 35 μm. The data were recorded by testXpert II software. For simplicity, only one representative strain–stress curve for each sample is shown in the manuscript. The tensile strength and elongation-at-break were obtained by averaging the values from three strain–stress curves for each sample.

### Morphological Observation of Injuries

The heating-induced restructuring of the MCP film was observed under an optical microscope (ZEISS SteREO Discovery. V20) with a 40× objective. The SEM observation on the surface morphology of the damaged samples was performed at the electron microscopy center of Empa. Punctured samples were prepared by stamping a needle through the surface of the film. Prior to measuring, the samples were coated with carbon by a Safematic CCU-010 coating machine. The sputtering time was 50 s, and the thickness of the coating was 10 nm. The cross-sectional morphology and elemental distribution spectroscopy of the MCP film were recorded by a field-emission scanning electron microscope (FEI Quanta FEC 650, Germany). The FE-SEM measurement was carried out at a relatively low accelerating voltage of 0.5 kV with an Everhart–Thornley detector.

### Transmission Electron Microscopy

The water dispersion of Fe_3_O_4_ NPs was diluted ten times and nebulized on a standard copper TEM grid covered with an amorphous carbon film. The measurements were carried out in a JEOL JEM-2200FS transmission electron microscope, which was equipped with a field emission gun operating at 200 kV with an ultra-high-resolution objective lens and with a highly sensitive 2k × 2k CCD camera (Gatan, Inc., USA). The TEM images were analyzed by ImageJ v1.8.0 software.

### Small-Angle X-ray Scattering

The SAXS experiments were carried out at the P03 MiNaXS beamline at the synchrotron source PETRA III at Deutsches Elektronen Synchrotron (DESY), Germany.^42^ The experiments were performed at an energy of 11.8 keV with a wavelength of λ = 0.105 nm. The beam size was 80 × 56 μm^2^ (horizontal × vertical). The sample–detector distance was kept at 9570 ± 5 mm. A Pilatus 2 M detector (Dectris AG, Switzerland) with a pixel size of 172 × 172 μm^2^ was used for SAXS. SAXS measurements were conducted with a heating chamber mounted onto an in situ tensile device.^43^ The heat flow was created by an external power supply, and the heating temperature was increased steadily from 30, 40, 50, 60, 70 to 80 °C and then decreased to 30 °C.

### X-ray Scattering Analysis

To obtain a quantitative analysis of the structural information, azimuthal cuts of the two-dimensional SAXS data were made by DPDAK v 1.4.1 software^51^ in the indicated region from *q* = 0.03 to *q* = 0.7 nm^–1^. The calculation of the scattering length density (SLD) values of Fe_3_O_4_ NP, Si-CNC, and PDMS was conducted by the calculating tool from the Website of https://www.ncnr.nist.gov/. The calculated SLD values were 40.4 × 10^–6^/Å^2^, 15.5 × 10^–6^/Å^2^, and 9.1 × 10^–6^/Å^2^ for Fe_3_O_4_, Si-CNC, and PDMS, respectively. All modeling of the SAXS curves was done using SASView v5.0.3 software,^52^ which is an open-source analysis software for small-angle scattering data. Four geometrical objects were used for fitting the data, including a core–shell sphere, a cylinder, a sphere with uniform scattering length density, and a hard sphere, which calculates the interparticle structure factor for monodisperse spherical particles interacting through hard sphere interactions. The average structural parameters of these objects were extracted from SEM or TEM images by ImageJ version 1.8.0 software to restrict the flexibility of fitting. The χ^2^ values were used as an indicator for evaluating the suitable fits.

### X-ray Diffraction

Powder XRD patterns were obtained using a Panalytical X’Pert PRO X-ray diffractometer (Philips), equipped with a Johansson monochromator (Cu–Kα_1_ radiation source, 1.5406 Å) and an X’Celerator linear detector. The samples were prepared by drop-casting the aqueous dispersion of Fe_3_O_4_ NPs and the mixture of the functional components of the MCP film and allowing them to dry under ambient conditions (*T* = 25 °C, RH = 30%). The diffraction patterns were recorded between 20° and 70° (2θ) with an angular step interval of 0.0167°. The average crystallite size of the nanoparticles was calculated from the full width at half-maximum of the diffraction peak (311) by using the Scherrer eq (eq (1)).^30^ A large fwhm of 1.05° permits that the typical instrument broadening of the aforementioned diffractometer (0.05°) can be neglected. With a shape factor of *K* = 1, the crystallite size was calculated as 8.3 nm:
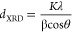
(1)

### Vibrating Sample Magnetometry

A Quantum Design VSM system operating at 7 T was used to measure the magnetic properties of the MCP film. The measurements were conducted at a temperature of 300 K under magnetic fields of up to 4 T.

### Dynamic Light Scattering

To measure the particle size distribution, DLS experiments were performed with the water dispersion of Fe_3_O_4_ NPs and the mixture of Fe_3_O_4_ NPs, Si-CNC, and PDMS using a Zetasizer NanoZS instrument (Malvern Instruments, UK) with Malvern Zetasizer v7.03 software for data analysis.

### Differential Scanning Calorimetry

Thermal analysis was conducted with a DSC device (PerkinElmer DSC 7, Germany). The samples were dried in a vacuum oven at a temperature of 25 °C for 24 h before testing. The tests were performed under nitrogen purge in a heat–cool–heat cycle at heating–cooling rates of 10 °C·min^–1^ from 0 to 120 °C. The results presented and discussed were taken from the second heating curve. Three samples of each material were measured and analyzed.

### UV/Vis Spectroscopy

The UV/vis spectra were recorded with a Czerny–Turner type CCD spectrometer CCS200 (Thorlabs GmbH, Germany), covering a wavelength range from 200 to 1000 nm with 3648 equidistant increments and a resolution better than 2 nm. A stabilized deuterium lamp (SLS204, Thorlabs GmbH, Germany) was used as the light source. The aqueous dispersions of Fe_3_O_4_ NPs and the MCP film were measured in a quartz cuvette, the holder of which was connected to the spectrometer and light source by two optical fibers. The dark current spectrum *I_d_* was obtained from the average of 100 spectra with 6 ms integration time when the detector was shielded from any light. The blank spectrum *I_b_* (deionized H_2_O reference in quartz cuvette) was obtained from the average of 100 spectra with 6 ms integration time. The sample spectrum was obtained by averaging 10000 spectra that were recorded with an integration time of 6 ms for the samples. The absorption spectra shown in Figure 6d were obtained as sample absorbance (ABS) spectra of sample spectra *I_s_* by eq (2):
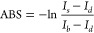
(2)

### Thermal-Assisted Welding

Thermal-assisted welding was achieved by a sequential treatment of overlapping two pieces of MCP films at the edges, heating at 70 °C for 20 min, and moderately pressing the overlapped region. The size of the overlapping region was 5 mm in length and 1 mm in width. The flower-shaped actuator devices were fabricated with the same procedure.

### Thermal-Assisted Remolding

An MCP and a Si-CNC/PDMS film with the same surface area (30 mm × 30 mm) were cut into pieces by scissors. The pieces of the two films were heated at 70 °C for 30 min under a pressure of 5 MPa. Silicon modules with the shapes of a butterfly and an owl were used for molding, respectively. The molded samples were transferred to an ambient condition and were carefully retrieved after being left at room temperature for 2 h.

### Modular Assembly of the MCP Cubes through Joining

The MCP films and Si-CNC/PDMS films were cut into square-shaped units (9 × 9 × 100 μm). The units were heated at the cut surfaces (in the thickness direction) of the films at 60 °C (Si-CNC/PDMS films) and 70 °C (MCP films) for 5 min to avoid melting, respectively. The units were joined together at the heated surfaces and then kept at the assembled states in a silicon mold and cooled down to room temperature (25 °C). Three different cubes were prepared, including cubes with joint interfaces composed of a homogeneous material (cube 1), cubes with joint interfaces composed of heterogeneous materials (cube 2), and cuboids with joint interfaces composed of heterogeneous materials (cube 3). Below are the geometries of the three types of cubes. Cube 1: six Si-CNC/PDMS units (9 × 9 mm × 100 μm); cube 2: two MCP units and four Si-CNC/PDMS units with the same size (9 × 9 mm × 100 μm); cube 3: four Si-CNC PDMS units and two MCP units. The bottom/top and front/back surfaces of the cube are Si-CNC/PDMS films with sizes of 9 × 18 × 100 and 16 × 18 × 100 μm, respectively. The sizes of the two MCP films were 9 mm × 16 mm × 100 μm. The “handbag” and “snowboard” in Figure S7 were prepared in a similar way. For fabricating the handbag, an open cube 3 and two strips of MCP films (3 × 30 mm × 100 μm) were used. The joint interfaces of the “handbag” were achieved through heating at the edges (3 mm × 5 mm) of the “belts” at 70 °C for 5 min, followed by cooling at room temperature for 30 min. The “snowboard” was fabricated from a Si-CNC/PDMS film (10 mm × 40 mm × 100 μm) and two MCP films (8 mm × 13 mm × 100 μm). The joint interfaces of the “snowboard” were achieved by heating the MCP films at the two edges of the bottom surface at 70 °C for 3 min, followed by cooling at room temperature for 30 min.

### Hierarchical Assembly

Cubes 2 and 3 were chosen to demonstrate hierarchical assembly functions. The joint surfaces of cubes 2 and 3 were heated at 70 °C for 5 min and then stacked together at the heated interfaces. The cubes in the assembled states were protected with a silicon mold and cooled down until they reached room temperature (25 °C).

### Sealing Functions

A water-soluble blue ink was used as the indicator to demonstrate the two modes of liquid loading strategies. In mode 1, the blue solution (400 μL) was injected with a syringe needle through the top surface of a closed cube 2. The cube was heated with a hot metal bar (60 °C) at the injection point of the cube for 10 min and was placed for 30 min at room temperature. In mode 2, the blue solution (400 μL) was dropped with a pipet from the top of an open cube 2. The cube was subsequently sealed with a Si-CNC/PDMS film (9 mm × 9 mm × 100 μm) already heated at 60 °C for 5 min, the cap was held for 10 min, and then, the cube was allowed to stabilize at room temperature for 30 min.

### Liquid-Releasing Function

Cube 2 (mode 1) was placed into a beaker filled with 10 mL of DI H_2_O with a tweezer at room temperature (25 °C). The beaker was then heated at 70 °C for 35 min. After cube 2 began to melt, the blue solution started to leak out. The beaker was transferred away from the heater, and the blue aqueous medium was carefully removed from the beaker. The remaining melts of the films in cube 2 were placed at room temperature for 96 min.

### Solid-Encapsulating Function

An open cube 3 was used as the container, and a metal stamp (6 × 14 × 15 mm, 11.1 g) was used as the indicator. The stamp was loaded into cube 3, which was then sealed in mode 2. The sealed cube 3 was placed into a beaker filled with 10 mL of DI H_2_O, which was subsequently heated at 70 °C for 30 min. After cube 3 began to melt, the beaker was moved away from the heater, and the water was removed out of the beaker with a pipet. The remaining melts of the films in cube 3 were placed at room temperature for 4 h. When the melts of cube 3 solidified, we obtained the mirrored-symmetrical logo of the stamp.

### Infrared Imaging

Temperature profiles on the MCP film were captured by an infrared camera (ImageIR 8300 hp, Infratec AG.). The radiation detector is an InSb quantum detector that is sensitive in the 2–5.7 μm wavelength range. During the measurements, the IR camera recorded up to 200 frames per second with a resolution of 640 × 512 pixels at 0.149 mm·pixel^–1^. The emissivity, transmittance, and reflectivity of the MCP film were measured. Films with dimensions of 8 mm × 20 mm (width × length) were fixed by two clips and irradiated by LEDs with different wavelengths at the same current density of 89 mW·cm^–2^ until reaching their maximum temperature, and the LED was then switched off. The mountable LEDs were purchased from Thorlabs GmbH, Germany. An IR camera was used to record the temperature variation during this process. The highest temperature was obtained under the illumination of an IR LED (wavelength 780 nm). We further measured the temperature of the samples under IR LEDs at increasing power densities of 2.5, 7.8, 13.4, 38, 68, and 89 mW·cm^–2^, respectively.

## Supporting Information Available

FTIR spectrum of the MCP film; cross-sectional SEM micrographs; EDS analysis for Fe_3_O_4_ NPs in the MCP film; magnetic hysteresis curve of the MCP film; remolding of the MCP films with a heating–cooling cycle; schematics and photographs for assembling cube 1; photograph and conceptual scheme of a handbag and snowboard; sealing and releasing functions of the modularly assembled MCP containers; photo of the beamline setup for temperature-controlled SAXS measurements; scan procedure of the SAXS measurement for the MCP film; one-dimensional SAXS integration of the punctured MCP film; example of the fit report of the MCP film at 30 °C; radii of the core-shell sphere collected at varying temperatures; thickness of the core-shell sphere collected at varying temperatures; radii of the cylinder collected at varying temperatures; length of the cylinder collected at varying temperatures; schematic of the experiment setup of IR camera imaging; time-dependent variation of the temperature recording of the MCP film; heating-induced transition points of the microstructural changes, heat absorption ability, and viscoelastic properties of the MCP film; and emissivity, transmittance, and reflectivity of the MCP film (PDF)
Magnetoresponse of the actuator based on a thermally assisted welded MCP film (avi)
Magnetoresponse of a flower-shaped MCP film after thermal-assisted welding (avi )
Infrared camera recording the photothermal response of the M layer (avi )

## Supplementary Material

nn4c14311_si_001.pdf

nn4c14311_si_002.avi

nn4c14311_si_003.avi

nn4c14311_si_004.avi

## Abbreviations

PNC: polymer nanocomposites
NP: nanoparticle
PDMS: polydimethylsiloxane
Si-CNC: silylated cellulose nanocrystal
MCP: magnetite nanoparticle/Si-CNC/PDMS
SEM: scanning electron microscopy
DLS: dynamic light scattering
SAXS: small-angle X-ray scattering
VSM: vibrating sample magnetometry
DMA: dynamic mechanical analysis
DSC: differential scanning calorimetry
XRD: X-ray diffraction
FTIR: Fourier transform infrared spectroscopy
