# Supplementary material for: Responsive Magnetic Polymer Nanocomposites through Thermal-Induced Structural Reorganization
Source: ACS Nano. 2025 Feb 6;19(6):6165–79. doi: 10.1021/acsnano.4c14311 (PMC11841046; doi:10.1021/acsnano.4c14311)
Supplement: Supplementary file 1 — nn4c14311_si_001.pdf [file nn4c14311_si_001.pdf]

Supporting information

Responsive magnetic polymer nanocomposites through thermal-induced structural reorganization

Qing Chen[a,b,c,]*, *Roman Furrer*[d], *Loghman Jamilpanah*[c e], *Andrei Chumakov*[f], *Yusuf Bulut*[f,g], *Constantin Harder*[f], *Peter Müller-Buschbaum*[g], *Stephan V. Roth*[f,h,]*, and *Artur Braun*[c,]*

[a]Spallation Neutron Source Science Center, 523803 Dongguan, China

[b]Institute of High Energy Physics, Chinese Academy of Science, 100049 Beijing, China

[c]Laboratory for High Performance Ceramics, Empa, Swiss Federal Laboratories for Materials Science and Technology, 8600 Dübendorf, Switzerland

[d]Transport at Nanoscale Interfaces Laboratory, Empa, Swiss Federal Laboratories for Materials Science and Technology, 8600 Dübendorf, Switzerland

[e]Magnetic and Functional Thin Films Laboratory, Empa, Swiss Federal Laboratories for Materials Science and Technology, 8600 Dübendorf, Switzerland

[f]Deutsches Elektronen-Synchrotron, 22607 Hamburg, Germany

[g]Technical University of Munich, TUM School of Natural Sciences, Department of Physics, Chair for Functional Materials, 85748 Garching, Germany

[h]Department of Fiber and Polymer Technology, KTH Royal Institute of Technology, 10044 Stockholm, Sweden

Corresponding authors * chenqing@ihep.ac.cn (Q. Chen);

* svroth@kth.se (S. V. Roth);

* artur.braun@alumni.ethz.ch (A. Braun)

1. Supplementary figures and tables

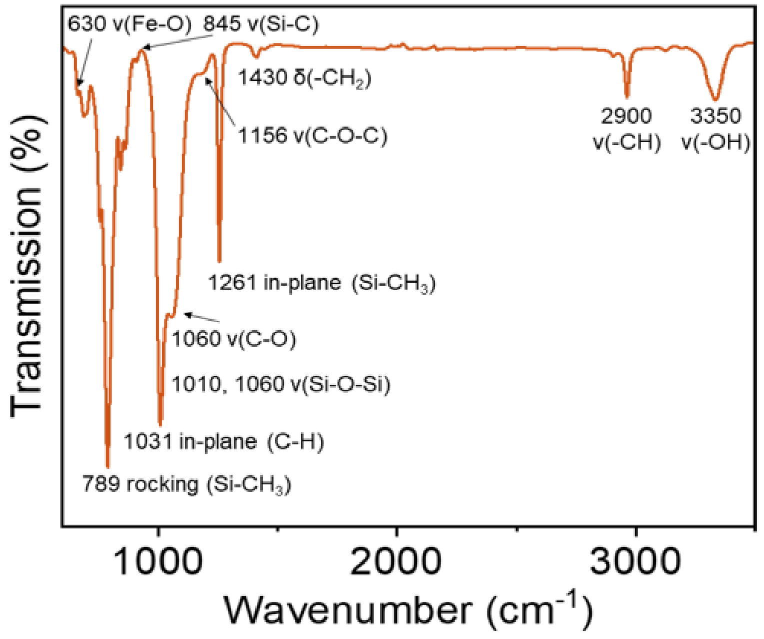

Figure S1. FTIR spectrum of the MCP film.

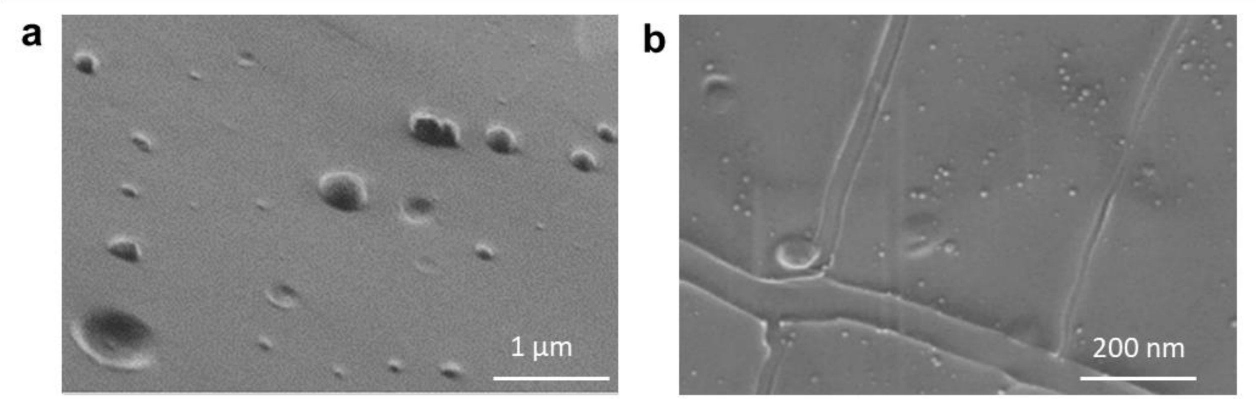

Figure S2. Cross-sectional SEM micrographs of the MCP film at a lower magnification to visualize the relatively large Fe_3O_4 NP assemblies (a), and at a higher magnification to visualize the small Fe_3O_4 NP clusters (b).

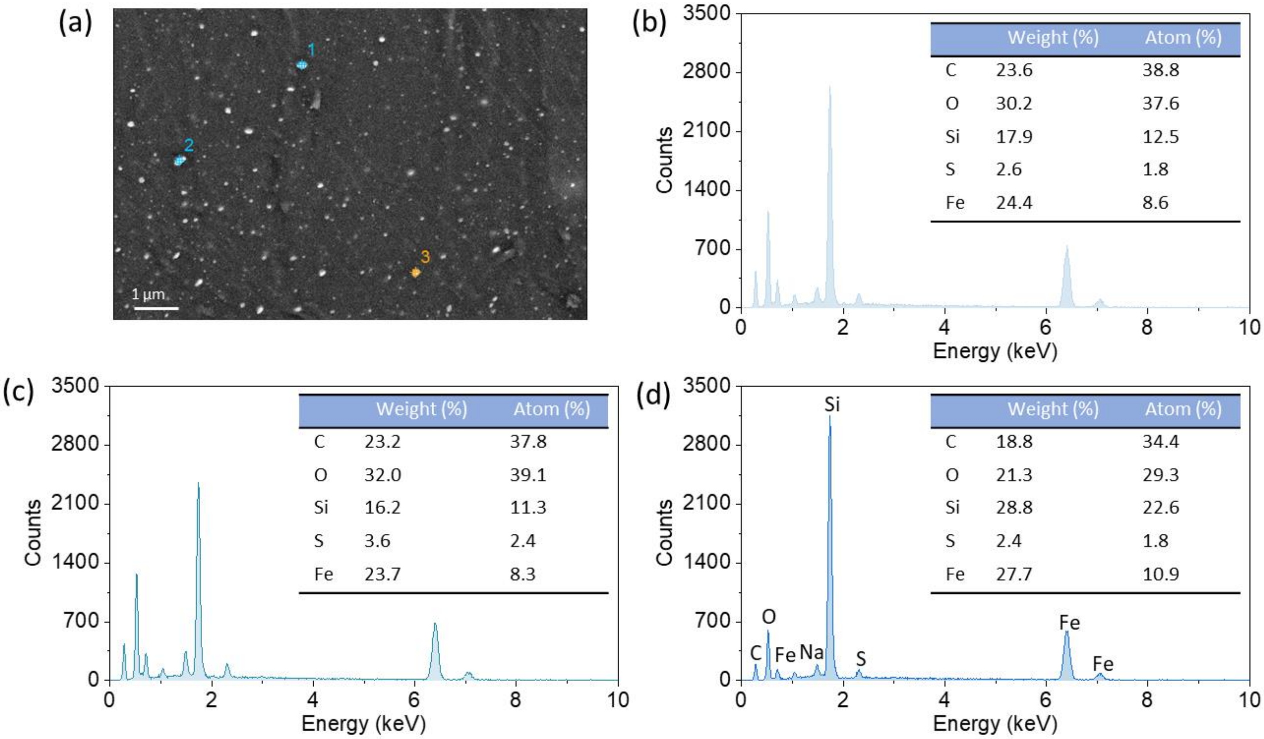

Figure S3. EDS analysis for Fe3O4 NPs in the MCP film. (a) FE-SEM image showing the three sample positions used for EDS analysis. (b-d) The EDS spectrum of the three bulges performed at the sample positions 1 (b), 2 (C) and 3 (d), respectively.

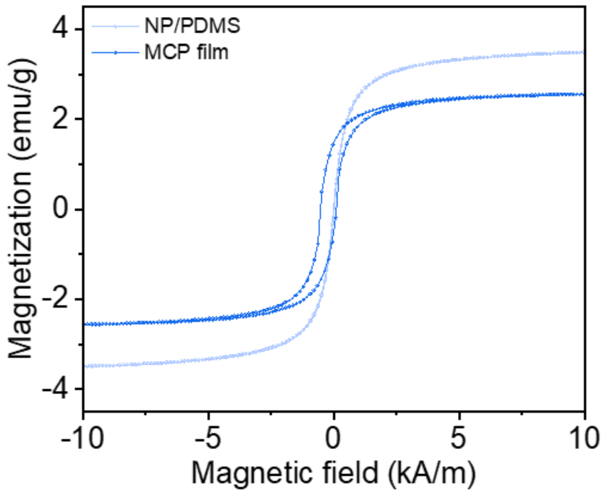

Figure S4. Magnetic hysteresis curve of the MCP film, compared to the superparamagnetic Fe_3O_4 NP/PDMS film measured at 25 °C.

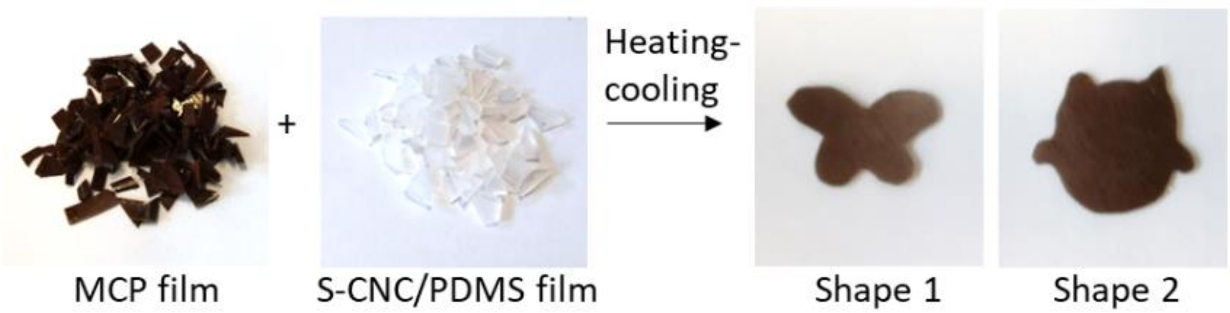

Figure S5. Remolding of the MCP films with a heating-cooling cycle.

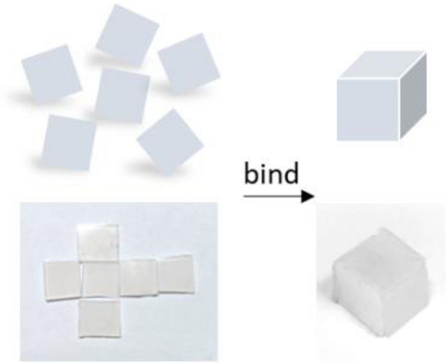

Figure S6. Schematics and photographs for Assembling of Cube 1 (Si-CNC/PDMS films).

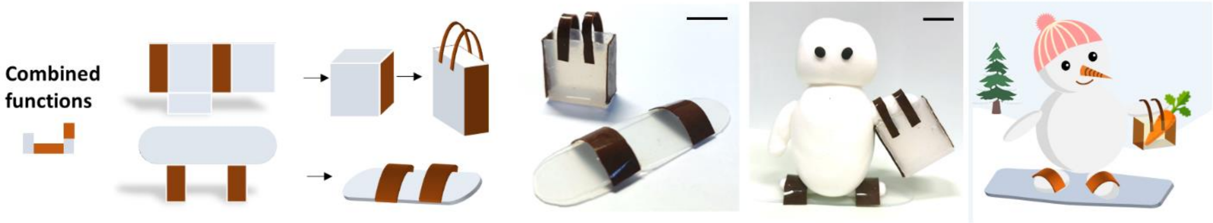

Figure S7. Photograph and conceptual scheme of a handbag and snowboard assembled by welding/healing of the MCP and Si-CNC/PDMS films for skiing activities. The scale bar is 10 mm.

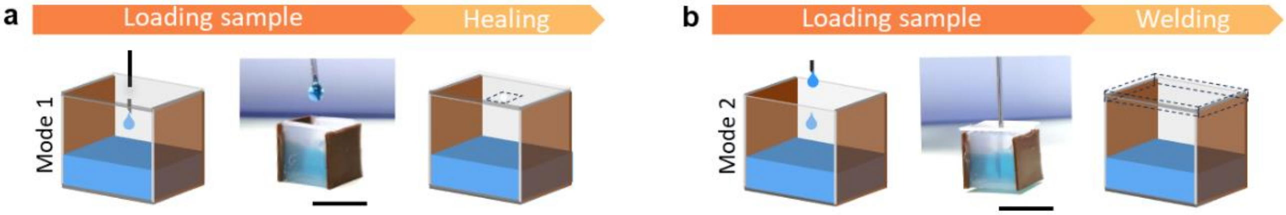

Figure S8. Sealing and releasing functions of the modularly-assembled MCP containers. (a, b) Loading liquid samples into the MCP containers (Cube 2) by injection and healing (a, mode 1). or by dropping and welding (b, mode 2).

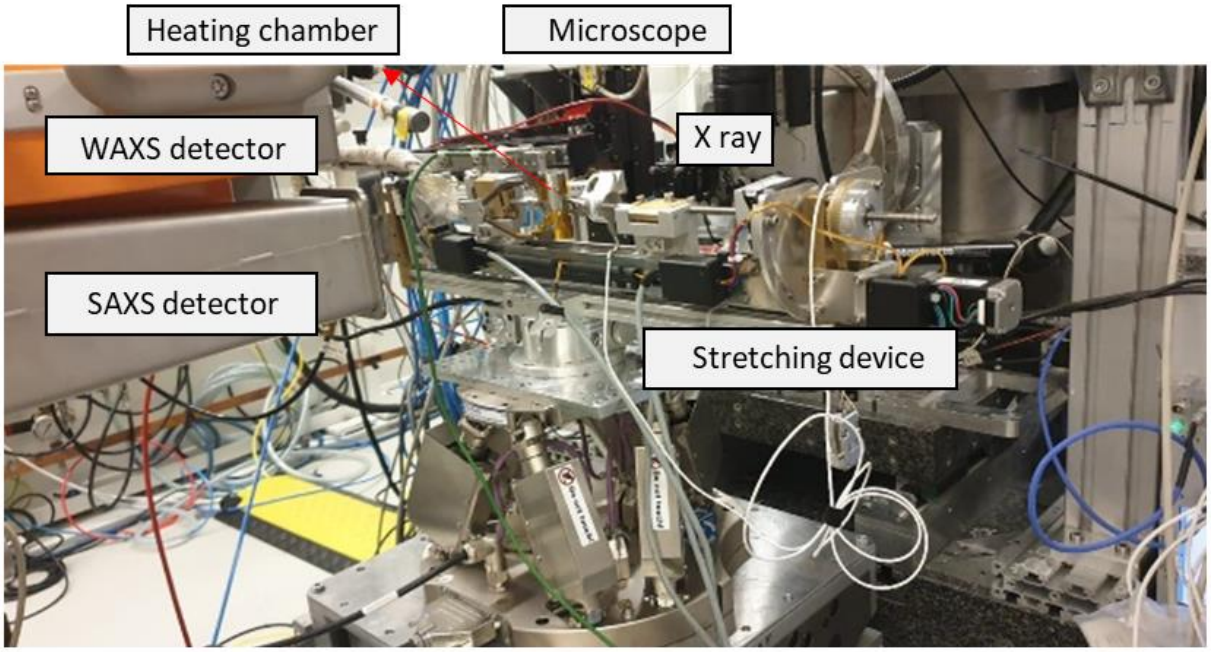

Figure S9. Photo of the beamline setup for temperature controlled SAXS measurements which incorporates an *in situ* stretching device as the sample holder and a heating chamber.

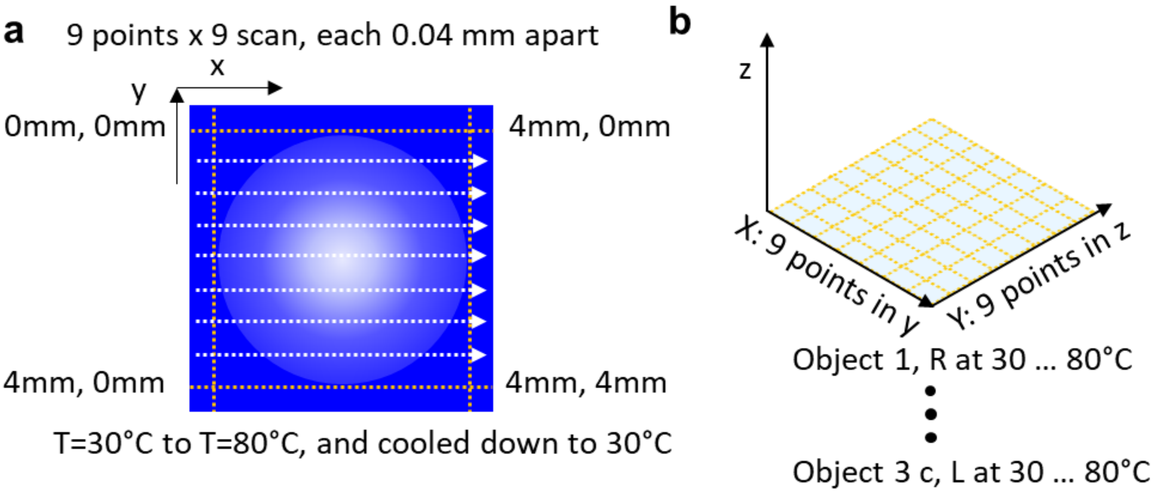

Figure S10. (a) Scan procedure of the SAXS measurement for the MCP film, which runs in both q_y and q_z directions at each temperature step. (b) Display of fitting parameters of the MCP film,

with the x and y axes in Figure 4d-i corresponding to the scan position in q_y and q_z scan directions, and z axis correspond to the values of fitting parameters, respectively.

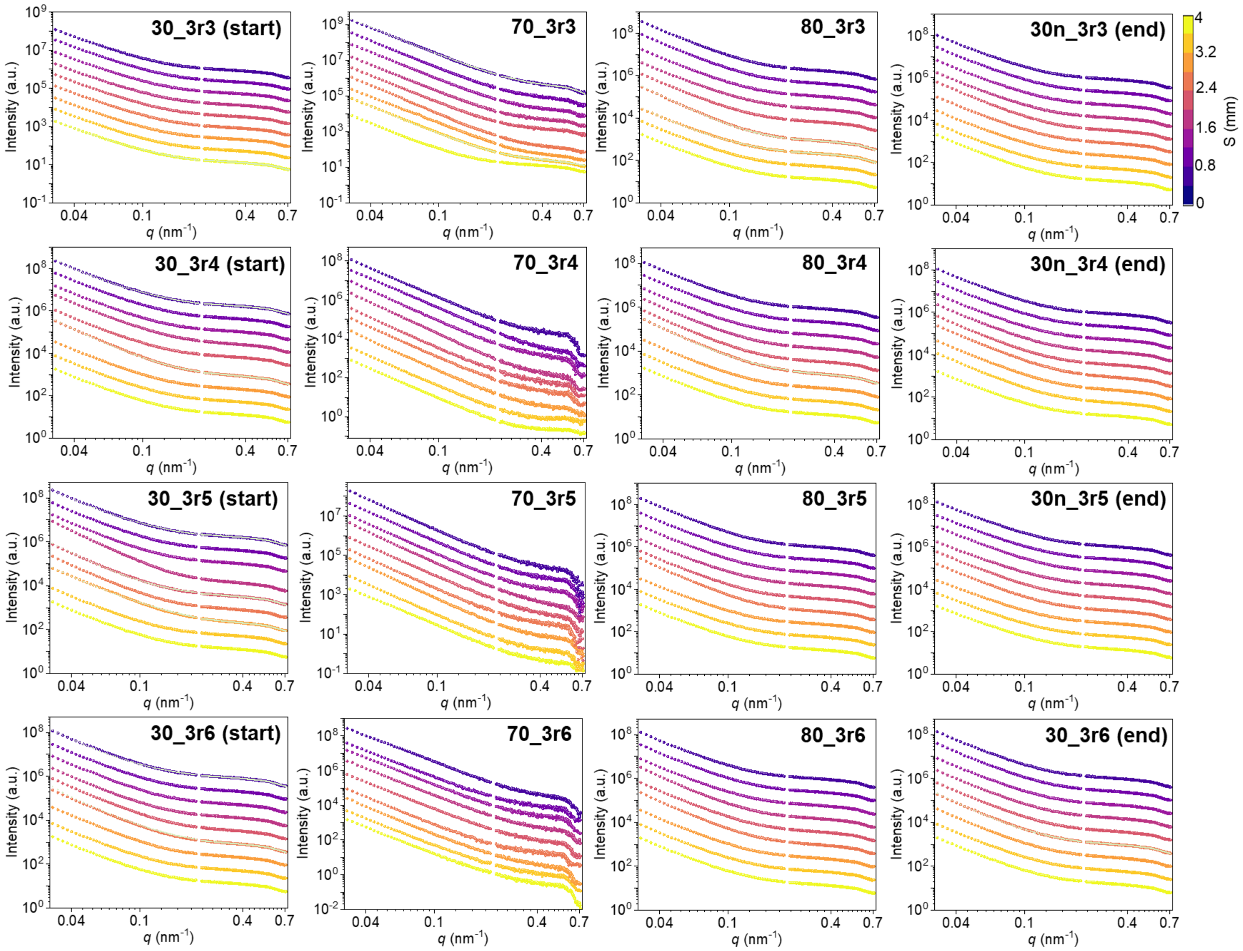

Figure S11. One-dimensional SAXS integration of the punctured MCP film at various scan positions as temperature increases from 30 to 80 °C, and then cooling down back to 30 °C.

(a)

File name: S24_30_1r7_00001_1.txt
SasView version: 5.0.3
SasModels version: 1.0.5
Fit optimizer used: Levenberg-Marquardt
Model name: core_shell_sphere+cylinder+sphere
Q Range: min = 0.03107855, max = 0.7111335
Chi2/Npts: 21.68
scale = 1.2 (fixed)
background = 7 (fixed) cm^{-1}
corshesphcorshecylsph = (fixed)
A_scale = 1.7 (fixed)
A_radius = 65 (fixed) Å
A_thickness = 46 (fixed) Å
A_sld_core = 40.4 (fixed) 10^{-6}/Å^2
A_sld_shell = 9.1 (fixed) 10^{-6}/Å^2
A_sld_solvent = 0 (fixed) 10^{-6}/Å^2
B_scale = 0.4 (fixed)
B_sld = 15.5 (fixed) 10^{-6}/Å^2
B_sld_solvent = 0 (fixed) 10^{-6}/Å^2
B_radius = 145 (fixed) Å
B_length = 310 (fixed) Å
C_scale = 0.7 (fixed) Å
C_sld = 40.4 (fixed) 10^{-6}/Å^2
C_sld_solvent = 0 (fixed) 10^{-6}/Å^2
C_radius = 4.3 (fixed) Å
Distribution of A_radius = 0.1 (fixed) Function: gaussian
Distribution of A_thickness = 0.05 (fixed) Function: gaussian
Distribution of A_length = 0.05 (fixed) Function: gaussian
Distribution of B_radius = 0.06 (fixed) Function: gaussian
Distribution of B_length = 0.1 (fixed) Function: gaussian
Distribution of C_radius = 0.01 (fixed) Function: gaussian

(b)

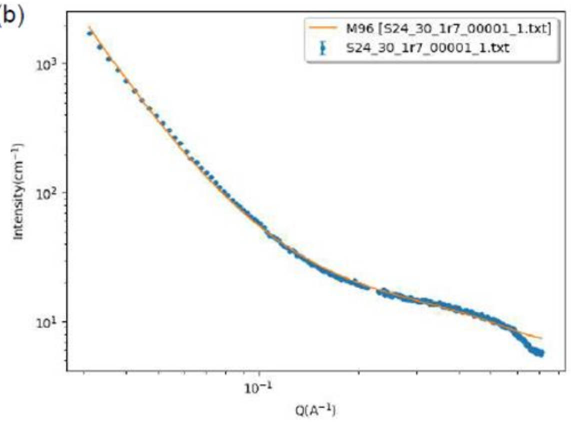

Figure S12. Example of the fit report of the MCP film at 30 ºC (start) at the scan position of *1r7* including (a) the exported fit parameters and (b) the fitted curve.

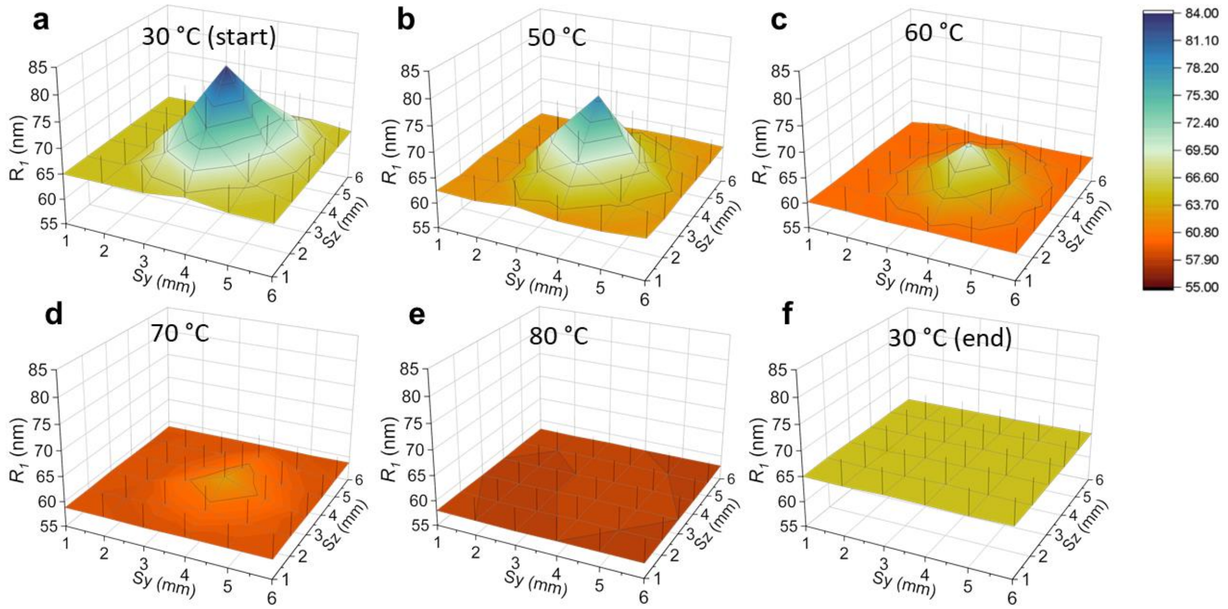

Figure S13. Radii of the core-shell sphere collected at varying temperatures and obtained from the SAXS fits.

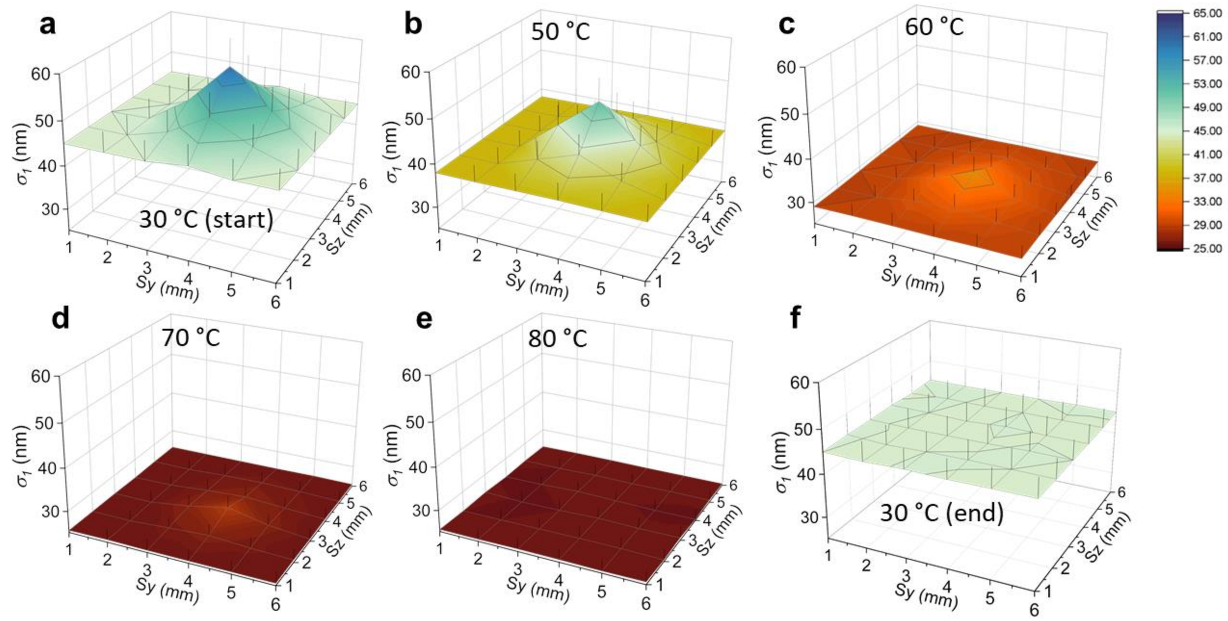

Figure S14. Thickness of the core-shell sphere collected at varying temperatures and obtained from the SAXS fits.

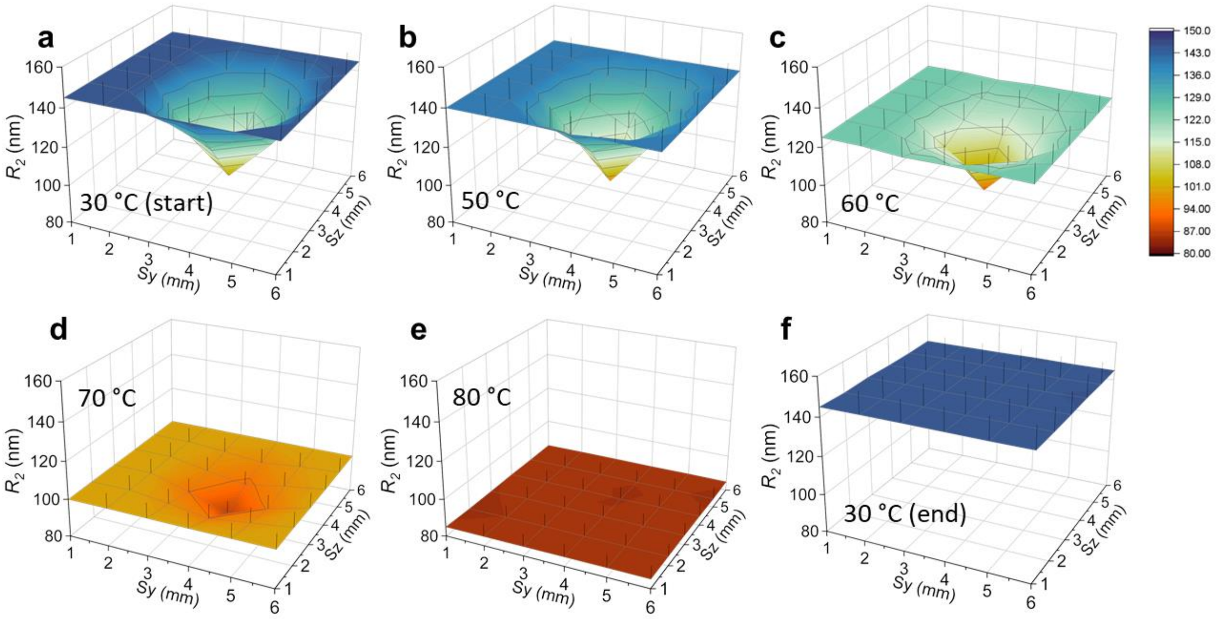

Figure S15. Radii of the cylinder collected at varying temperatures and obtained from the SAXS fits.

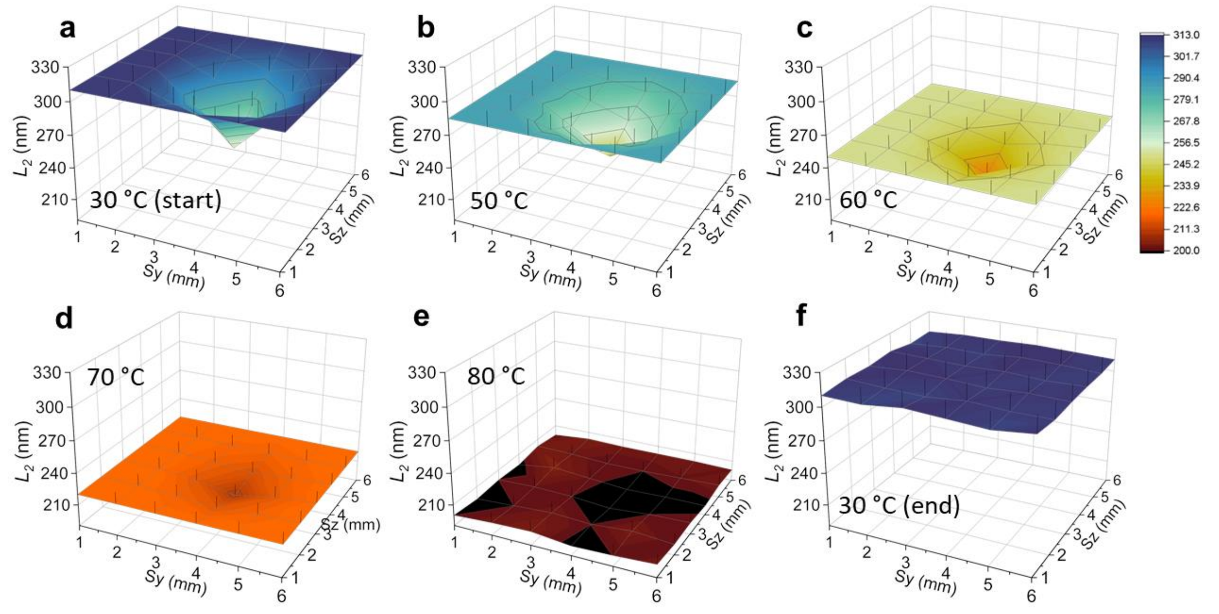

Figure S16. Length of the cylinder collected at varying temperatures and obtained from the SAXS fits.

Table S1. Heating-induced transition points of the microstructural changes, heat absorption ability, and viscoelastic properties of the MCP film detected by three analytical techniques with temperature control, including SAXS, DMA, and DSC, respectively.

Technique	T_{trans}	Indicator
**USAXS**	50 °C	Size of microstructures
**DMA**	50 °C	Plasticity, elasticity
**DSC**	70 °C	Heat absorption

Table S2. Emissivity, transmittance and reflectivity of the MCP film.

Emissivity ε	Transmittance τ	Reflectivity ρ
0.66	0.0	0.34

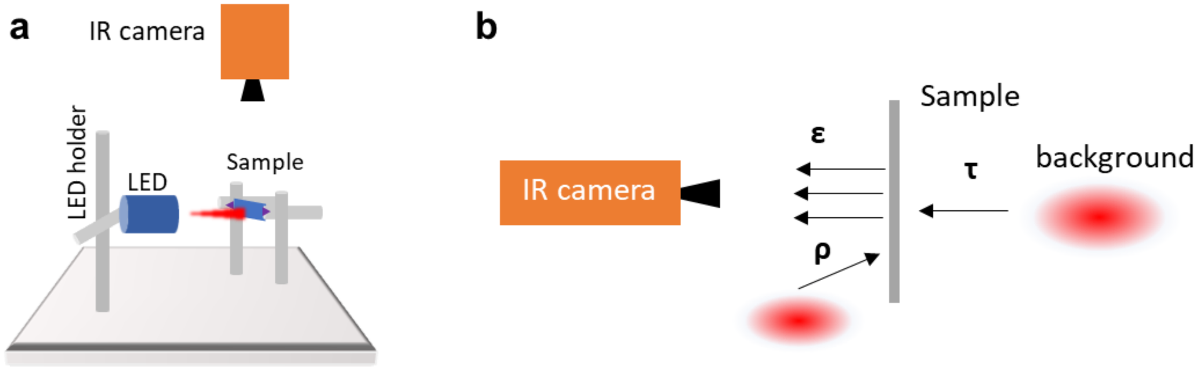

Figure S17. (a) Schematic of the experiment setup of IR camera imaging. (b) Schematic illustration of temperature calibration.

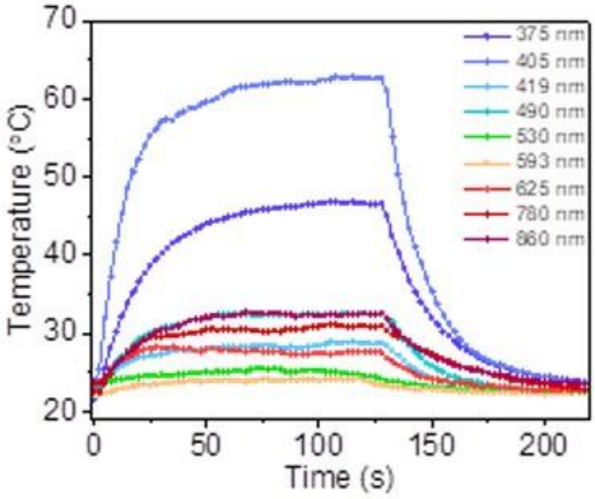

Figure S18. Time-dependent variation of the temperature recording of the MCP film when illuminated by LEDs at different wavelengths.

2. List of supporting movies

Movie S1. Magneto-response of the actuator based on a thermally-assisted welded MCP film. In this movie, the movement of the flower-shaped MCP actuator in response to an approaching magnetic stirrer bar retriever was recorded.

Movie S2. Magneto-response of a flower-shaped MCP film after thermal-assisted welding. In this movie, one could see the clockwise rotation of an MCP actuator when being placed upon a rotating magnetic field (with a water container upon a magnetic stirring machine).

Movie S3. Infrared camera recording the photo-thermal response of the M layer. This movie recorded the increase of the surface temperature of the M layer when the IR LED is switched on and the decrease of temperature when the IR LED is switched off.

3. Supplementary note 1

We fabricate a closed hollow cube (Cube 1) by joining six square-shaped units of the planar Si-CNC/PDMS films (9 mm × 9 mm × 100 μm, **Figure S6**) at their edges. The units with the homogeneous material composition (Si-CNC/PDMS matrix in this case) are gently heated at 60 °C for 5 minutes and kept at the assembled state during the cooling process to maintain its 3D shape. After cooling to room temperature, the hollow cube is retrieved. Another two types of hollow cubes are assembled by joining four square-shaped units of the planar Si-CNC/PDMS films together with two units of MCP films (**Figure 2h**). These two cubes with inhomogeneous material composition were fabricated with the same procedure as Cube 1 except that the MCP films are heated at 70 °C for 5 minutes at the edges before joining with the other units. The joining of Cube 2 and 3 are performed with the following procedure: Cubes 2 are heated gently at 60 °C for 5 minutes at the bottom surface (Si-CNC/PDMS unit), and then stacked together at the heated surfaces of Cube 3 in series.

4. Supplementary note 2

The liquid sealing function of the MCP containers (Cube 2) is performed by two modes of sample loading. An aqueous solution of blue ink (Finecolor®) is chosen as the aqueous medium to visualize the sealing effectivity of the MCP container. In mode 1, 400 μL blue solution is injected with a syringe needle into a closed Cube 2 through the Si-CNC/PDMS units (top surface). The punctured hole is subsequently healed by local heating after the needle is retrieved (**Figure S8a**). In mode 2, 400 μL blue solution is loaded dropwise with a syringe from the top of an open Cube 2, which is subsequently sealed with an Si-CNC/PDMS film (9 mm × 9 mm × 100 μm) already heated at 60 °C for 5 minutes (**Figure S8b**). On the other hand, solid objects are loaded only by mode 2.

To demonstrate the liquid sealing and releasing functions, a sealed Cube 2 (mode 1) filled with 800 μL blue solution is placed inside a glass beaker containing 10 mL of DI H_2O (25 °C). After 90 minutes of water immersion, Cube 2 maintains an intact 3D structure without leakage of the blue solution, indicating that the sealing is effective (**Figure 3a, ii**). These experiments imply that healing is an effective strategy of sealing the MCP cubes, which further enables them to be used as capsules for protecting chemicals from the ambient environment. Due to the thermo-responsive restructuring process of the MCP film, we deduce that the shape of the container can be reconfigured on demand to fit the needs of application. To test this possibility, the beaker is heated at a temperature of 70 °C to induce the shape deformation of MCP films. After 35 minutes, we observe that the components of the Cube 2 disassemble and sink to the bottom of the beaker (**Figure 3a, iii**). Meanwhile, the blue solution leaked out, and the liquid in the beaker turns into blue. After removing the aqueous medium from the beaker, the melted components of the MCP film are allowed to cool down at the ambient environment, and it recovers to a solid film with fixed

shape after 96 minutes (**Figure 3a, iv and v**). This phenomenon indicates the potential of the MCP films as customizable and reusable containers for controlled release of chemicals (**Figure 3b**).

To demonstrate the solid encapsulation functions of the MCP containers, we use a metal stamp (6 mm × 14 mm × 15 mm, 11.1 g) with the logo “Empa” as the cargo and place it into a hollow Cube 3 (9 mm × 18 mm × 18 mm). The cube is subsequently sealed with an Si-CNC/PDMS cap (9 mm × 18 mm × 100 µm) already heated at 60 °C for 5 minutes by mode 2. The cube is cooled down at room temperature and is transferred into a glass beaker filled with 10 mL of DI H_2O. The entire system, including the beaker, water, and the encapsulated stamp is heated at 70 °C. Ater 30 minutes of heating, we observe the disintegration of the components of the MCP films and the deformation of the cube. As a result, the metal stamp falls down to the bottom of the beaker, surrounded by the components of the melted MCP film. The aqueous medium is gently removed from the cube, which is further allowed to cool down to room temperature for 4 h for the components of the MCP film to solidify (**Figure 3c**). Finally, the stamp is gently released from the reformed MCP film, and as expected, it preserves the mirror-symmetric version of the “Empa” logo of the stamp. This experiment indicates that, in spite of reconfiguring their shapes into the desired mold geometry, the MCP containers can be used for transforming into adaptable devices with customizable shapes for flexible 3D fabrication (**Figure 3d**).
